# Reference proteins to improve Core 1 and Core 2 Alzheimer’s disease CSF and plasma biomarkers

**DOI:** 10.1093/brain/awaf375

**Published:** 2025-10-06

**Authors:** Linda Karlsson, Shorena Janelidze, Nicolas R Barthélemy, Kanta Horie, Joseph Therriault, Lorenzo Gaetani, Giovanni Bellomo, Suzanne E Schindler, Jacob Vogel, Ida Arvidsson, Kalle Åström, Brian A Gordon, Cyrus A Raji, Tammie L S Benzinger, John C Morris, Johanna Nilsson, Ann Brinkmalm, Sebastian Palmqvist, Erik Stomrud, Gemma Salvadó, Alexa Pichet Binette, Massimiliano Di Filippo, Lucilla Parnetti, Pedro Rosa-Neto, Kaj Blennow, Randall J Bateman, Niklas Mattsson-Carlgren, Oskar Hansson

**Affiliations:** Clinical Memory Research Unit, Department of Clinical Sciences in Malmö, Lund University, Lund SE-22184, Sweden; Clinical Memory Research Unit, Department of Clinical Sciences in Malmö, Lund University, Lund SE-22184, Sweden; Department of Neurology, Washington University School of Medicine, St. Louis, MO 63108, USA; The Tracy Family SILQ Center, Washington University School of Medicine, St. Louis, MO 63108, USA; Department of Neurology, Washington University School of Medicine, St. Louis, MO 63108, USA; The Tracy Family SILQ Center, Washington University School of Medicine, St. Louis, MO 63108, USA; Eisai Inc., Nutley, NJ 07110, USA; Translational Neuroimaging Laboratory, McGill University Research Centre for Studies in Aging, McConnell Brain Imaging Centre (BIC), Montreal Neurological Institute, Montreal Neurological Institute-Hospital, Montreal, Quebec H4H 1R3, Canada; Section of Neurology, Department of Medicine and Surgery, University of Perugia, Perugia 06132, Italy; Section of Neurology, Department of Medicine and Surgery, University of Perugia, Perugia 06132, Italy; Department of Neurology, Washington University School of Medicine, St. Louis, MO 63108, USA; Department of Clinical Sciences, SciLifeLab, Lund University, Lund SE-22184, Sweden; Centre for Mathematical Sciences, Lund University, Lund SE-22362, Sweden; Centre for Mathematical Sciences, Lund University, Lund SE-22362, Sweden; Department of Radiology, Washington University School of Medicine, St. Louis, MO 63130, USA; Department of Radiology, Washington University School of Medicine, St. Louis, MO 63130, USA; Department of Radiology, Washington University School of Medicine, St. Louis, MO 63130, USA; Department of Neurology, Washington University School of Medicine, St. Louis, MO 63108, USA; Department of Psychiatry and Neurochemistry, Institute of Neuroscience and Physiology, the Sahlgrenska Academy, University of Gothenburg, Mölndal SE-43180, Sweden; Department of Psychiatry and Neurochemistry, Institute of Neuroscience and Physiology, the Sahlgrenska Academy, University of Gothenburg, Mölndal SE-43180, Sweden; Clinical Memory Research Unit, Department of Clinical Sciences in Malmö, Lund University, Lund SE-22184, Sweden; Memory Clinic, Skåne University Hospital, Malmö SE-20502, Sweden; Clinical Memory Research Unit, Department of Clinical Sciences in Malmö, Lund University, Lund SE-22184, Sweden; Memory Clinic, Skåne University Hospital, Malmö SE-20502, Sweden; Clinical Memory Research Unit, Department of Clinical Sciences in Malmö, Lund University, Lund SE-22184, Sweden; Barcelonaβeta Brain Research Center (BBRC), Pasqual Maragall Foundation, Barcelona 08005, Spain; Clinical Memory Research Unit, Department of Clinical Sciences in Malmö, Lund University, Lund SE-22184, Sweden; Department of Physiology and Pharmacology, Université de Montréal, Montréal, Quebec H3T 1J4, Canada; Centre de Recherche de L’Institut Universitaire de Gériatrie de Montréal, Montréal, Quebec H3W 1W5, Canada; Section of Neurology, Department of Medicine and Surgery, University of Perugia, Perugia 06132, Italy; Section of Neurology, Department of Medicine and Surgery, University of Perugia, Perugia 06132, Italy; Translational Neuroimaging Laboratory, McGill University Research Centre for Studies in Aging, McConnell Brain Imaging Centre (BIC), Montreal Neurological Institute, Montreal Neurological Institute-Hospital, Montreal, Quebec H4H 1R3, Canada; Douglas Hospital Research Centre—Centre Intégré Universitaire de Santé et Services Sociaux de L’Ouest-de-L’Île-de-Montréal, Verdun, Quebec H4H 1R3, Canada; The Peter O’Donnell Jr. Brain Institute (OBI), University of Texas Southwestern Medical Centre (UTSW), Dallas, 75235 TX, USA; Department of Psychiatry and Neurochemistry, Institute of Neuroscience and Physiology, the Sahlgrenska Academy, University of Gothenburg, Mölndal SE-43180, Sweden; Clinical Neurochemistry Laboratory, Sahlgrenska University Hospital, Mölndal SE-43180, Sweden; Paris Brain Institute, ICM, Pitié-Salpêtrière Hospital, Sorbonne University, Paris 75013, France; Neurodegenerative Disorder Research Center, Division of Life Sciences and Medicine, and Department of Neurology, Institute on Aging and Brain Disorders, University of Science and Technology of China and First Affiliated Hospital of USTC, Hefei 230026, P.R. China; Department of Neurology, Washington University School of Medicine, St. Louis, MO 63108, USA; The Tracy Family SILQ Center, Washington University School of Medicine, St. Louis, MO 63108, USA; Clinical Memory Research Unit, Department of Clinical Sciences in Malmö, Lund University, Lund SE-22184, Sweden; Memory Clinic, Skåne University Hospital, Malmö SE-20502, Sweden; Clinical Memory Research Unit, Department of Clinical Sciences in Malmö, Lund University, Lund SE-22184, Sweden

**Keywords:** normalization, CSF biomarkers, plasma biomarkers, Aβ40, PET, Alzheimer’s disease

## Abstract

Concentration-based fluid biomarkers represent an informative and cost-effective way to detect and monitor Alzheimer’s disease (AD) pathology. However, non-AD-related interindividual variation in biofluids can also affect biomarker concentrations. Here, we investigated whether normalization of CSF and plasma biomarkers to reference proteins, such as amyloid-β40 (Aβ40) and non-phosphorylated mid-region tau (np-tau), improves their robustness and reliability of representing AD pathology load.

Using the Swedish BioFINDER-2 cohort [*n* = 1702, 50.7% male, mean (standard deviation) age 68.4 (12.2) years], we compared the associations between tau/Aβ-PET load and fluid biomarkers alone versus biomarkers in a ratio with a reference protein (Aβ40 or np-tau) in univariate linear regression models. Fluid biomarkers included CSF and plasma measures of p-tau217, p-tau181, p-tau205, np-tau181-190, np-tau195–210, np-tau212–221, Aβ42 and Aβ40; CSF MTBR-tau243, SNAP-25, neurogranin, YKL-40 and sTREM2; and plasma eMTBR-tau243. Biomarkers were measured with mass spectrometry assays and/or immunoassays. In addition, we performed validation and extended analyses, comparing, for example, group-level diagnostic differences and longitudinal biomarker trajectories, in three independent prospective cohorts [BioFINDER-1, Knight Alzheimer Disease Research Center (ADRC) and Translational Biomarkers in Aging and Dementia (TRIAD)] and in an Italian multiple sclerosis cohort.

CSF Aβ40 normalization significantly strengthened the associations of several core CSF AD biomarkers, including CSF MTBR-tau243, p-tau isoforms and synaptic biomarkers, with tau-PET (Δ*R*^2^ = 0.064–0.24) and Aβ-PET (Δ*R*^2^ = 0.016–0.28). Normalization to CSF np-tau mainly improved concordance with Aβ-PET (Δ*R*^2^ = −0.0059 to 0.19). The strongest association with tau-PET was observed for MTBR-tau243/Aβ40 (*R*^2^ = 0.78, compared with 0.65 for non-normalized MTBR-tau243), and with Aβ-PET for p-tau217/np-tau (*R*^2^ = 0.65, compared with 0.46 for non-normalized p-tau217). Plasma biomarker associations with tau-PET improved when using normalization to plasma Aβ40 or np-tau (Δ*R*^2^ = 0.004–0.14), with the strongest effect for eMTBR-tau243/np-tau (*R*^2^ = 0.72 versus 0.60). Associations with Aβ-PET were enhanced with np-tau normalization (Δ*R*^2^ = 0.018–0.16, strongest for p-tau217/np-tau: *R*^2^ = 0.62 versus 0.53). The results were replicated in Knight ADRC and TRIAD. Furthermore, longitudinal analyses showed that Aβ40 normalization typically reduced interindividual rather than intra-individual variability over time. Normalization did not enhance group-level differences in inflammatory CSF biomarkers in AD, nor did it improve biomarker associations in the multiple sclerosis cohort.

In conclusion, normalization of CSF and plasma biomarkers to reference proteins, such as Aβ40 or np-tau, enhances their association with brain tau and Aβ pathology, making already high-performing AD fluid biomarkers even more accurate.

## Teaser

Karlsson *et al.* show that normalizing Alzheimer’s disease cerebrospinal fluid and plasma biomarkers to reference proteins increases their agreement with tau- and amyloid PET, enhancing the accuracy of fluid biomarker tests for detecting pathological changes.

## Introduction

Alzheimer’s disease (AD) is characterized by the presence of amyloid-β (Aβ) plaques and tau neurofibrillary tangles in the brain, which can be detected with imaging biomarkers (e.g. PET) or fluid biomarkers (e.g. CSF or plasma).^1,2^ The recent development of effective disease-modifying therapies for AD has highlighted the importance of reliable and accessible diagnostic tools for AD.^3-5^ CSF biomarkers, measured as protein concentrations, represent an informative and cost-effective option and are clinically well established for AD.^1,6^ However, we showed previously that the average CSF protein abundance varies systematically across individuals, which can reduce the precision of CSF AD biomarkers compared with PET.^7^ To account for this interindividual signal, we identified reference proteins that can be used to normalize disease-specific CSF biomarkers and thereby improve their diagnostic performance. One such protein was CSF Aβ40, which was efficient for normalizing both CSF Aβ42 levels (as commonly done^8,9^) and CSF p-tau181 levels, congruent with results from Guo *et al*.^10^ However, it remains unclear what effect a reference protein such as CSF Aβ40 has on the relationship between other CSF AD biomarkers and the load of AD pathology. This is highly relevant in the context of the recently revised criteria for diagnosis and staging of AD, where several additional Core 1 (early-changing) and Core 2 (later-changing) AD fluid biomarkers were emphasized (e.g. MTBR-tau243, p-tau217 and p-tau205).^11^ It also remains unclear: (i) whether normalization to reference proteins reduces intra-individual CSF variability (e.g. for longitudinal monitoring of patients); (ii) whether the reference proteins are useful beyond AD biomarkers, such as for neuroinflammation and neurodegeneration markers; and (iii) whether plasma AD biomarkers can be improved by normalization to a reference protein in a similar manner to CSF biomarkers.

In this work, we aimed to evaluate the effects of Aβ40 normalization on the relationship between several AD-related CSF and plasma biomarkers with continuous tau- and Aβ-PET load, representing the level of tau and Aβ pathology in the brain, respectively. We compared the performance of Aβ40 with non-phosphorylated mid-region tau (np-tau), another commonly used normalization marker,^12,13^ and with Aβ42, which has been proposed as a ratio denominator in previous works.^14-18^ We also examined differences in longitudinal variability of raw versus normalized CSF and plasma biomarkers to investigate whether reference proteins could lead to increased biomarker robustness also within an individual over time. Lastly, we explored generalization properties of CSF Aβ40 normalization for inflammatory biomarkers in both AD and other neurological diseases.

## Materials and methods

### Participants

For the main analyses, we included two cohorts: the Swedish BioFINDER-2 (BF2) cohort (enrolment from 2017 and still enrolling, NCT03174938) and the Swedish BioFINDER-1 (BF1) cohort (enrolment between 2010 and 2015, NCT01208675). Both cohorts consisted of individuals with normal cognition (NC), subjective cognitive decline (SCD), mild cognitive impairment (MCI), AD dementia or another neurodegenerative disease. All participants were recruited at Skåne University Hospital and the Hospital of Ängelholm, Sweden. Both cohorts had biannual follow-up including longitudinal assessments over a period of 6–10 years. Additional detailed information about the cohorts has been described previously.^19,20^ The included participants had undergone tau-PET and/or Aβ-PET and had at least one of the relevant plasma or CSF biomarker measures available. For replication analyses, the prospective Alzheimer’s disease cohorts Charles F. and Joanne Knight-Alzheimer’s Disease Research Center (Knight ADRC) from Washington University, USA, and Translational Biomarkers in Aging and Dementia (TRIAD) from McGill University, Canada, were included; both cohorts also described previously.^12,21^ The cohorts had a study population similar to BF1 and BF2, with individuals spanning the cognitive spectrum from normal cognition to dementia and other neurodegenerative conditions and with measurements of relevant CSF and plasma biomarkers, in addition to Aβ- and/or tau-PET. TRIAD and a multiple sclerosis (MS) cohort from University of Perugia,^22^ Italy, were also used to explore generalization properties of reference protein normalization to neuroinflammatory markers. All data were collected between 2005 and 2023.

### Ethics

All participants gave written informed consent to participate. The studies were approved by the Regional Ethical Committee in Lund, Sweden (BF1 and BF2), the Washington University Human Research Protection Office (Knight ADRC), the Research Ethics Board Office of the McGill University (TRIAD) and the Ethics Committee of Regione Umbria (Perugia MS cohort).

### CSF and plasma collection and analysis

CSF and plasma samples were collected during the baseline clinical examination and handled according to established preanalytical protocols, previously described for all cohorts.^12,19,22-25^ All analyses were performed by technicians blinded to all clinical and imaging data. SNAP-25 was quantified at the University of Gothenburg (GU), using an in-house mass spectrometry assay.^26^ The other CSF and plasma biomarkers were measured with the Roche Diagnostics International Ltd Elecsys (El)/NeuroToolKit (NTK) assays,^27^ Eli Lilly assays on a Meso Scale Discovery platform (Li),^19,24^ mass spectrometry assays developed at Washington University (WU)^13,28,29^ or Proximity Extension Assays developed by OLINK Proteomics Uppsala, Sweden. Note that OLINK preprocessing included normalization for technical variation across plates, but did not include within-sample scaling or normalization across proteins (which could reduce reference protein normalization effects). CSF Aβ42/Aβ40 was used to define Aβ positivity according to previously established cut-offs of <0.08 in BF2,^30^ <0.066 in BF1^31^ and <0.0673 in Knight ADRC.^12^ For ratios involving phosphorylated tau (p-tau) and mid-region non-phosphorylated tau (np-tau) (also commonly referred to as %p-tau), the corresponding unmodified tau peptide, containing the amino acid residue of the p-tau marker, was used (e.g. np-tau212–221 for p-tau217). For MTBR-tau243, np-tau195–210 was used.

### PET imaging

Tau-PET was performed in BF2, Knight ADRC and TRIAD using ^18^F-RO948, ^18^F-flortaucipir and ^18^F-MK6240, respectively. Standardized uptake value ratio (SUVR) images were created for the 70–90 min (^18^F-RO948), 80–100 min (^18^F-flortaucipir) and 90–110 min (^18^F-MK6240) post-injection interval using the inferior cerebellar cortex as the reference region. Two composites, corresponding to (i) a Braak I–IV temporal meta-region of interest (ROI); and (ii) a Braak V–VI neocortical ROI, were used to represent earlier and later stages of continuous AD-related tau pathology.^32,33^ Aβ-PET was performed using ^18^F-flutemetamol in BF1 and BF2, ^18^F-florbetapir (^18^F-AV45) or ^11^C-PiB in Knight ADRC, and ^18^F-AZD4694 in TRIAD. Aβ-PET SUVR images were created for the 90–110 min (^18^F-flutemetamol), 50–70 min (^18^F-florbetapir), 30–60 min (^11^C-PiB) and 40–70 min (^18^F-AZD4694) post-injection interval, with whole cerebellum as the reference region. Given that two different tracers were used within Knight ADRC, these SUVR images were converted to centiloids.^34^ A global neocortical composite ROI was used to represent continuous Aβ-PET load.^35^ Aβ-PET was used to define Aβ positivity in TRIAD (SUVR > 1.55).^36^ Microglial activation *TSPO*  ^11^C-PBR28 PET imaging was performed in TRIAD, measuring uptake in the posterior cingulate cortex.^37^

### Statistical analysis

All analyses were performed using Python version 3.9 (code available at https://github.com/DeMONLab-BioFINDER/karlsson_refprot_normalization). Variables were standardized (*z*-scored) within each cohort. Two extreme outliers were removed in BF2: both cases were Aβ-positive individuals with plasma Aβ40 and Aβ42 values >10 standard deviations (SD) above the Aβ-positive group mean. For the main analyses, normalization was performed by calculating the ratio of the fluid biomarker (numerator) to the reference protein (denominator). As a sensitivity analysis, normalization was instead performed by adjusting for the reference protein as a covariate. Univariate linear regression models were used to compare continuous explained variance (*R*^2^) between unnormalized and normalized biomarkers and PET composites. Given that BF2 had a varying number of individuals with both fluid biomarker data and PET, we first used all available data for each biomarker (reporting the *n* for each model in the corresponding results tables) and second compared biomarkers across the subsample of participants with all data available. For group comparisons, we used logistic regression models and evaluated the area under the receiver operating characteristic curve (ROC AUC). Gaussian mixture modelling was used to binarize outcome variables. For longitudinal biomarker concentration, we used linear mixed-effects models with random intercepts for each participant (no random slopes, because many individuals had only two longitudinal measures available). These models also included Aβ positivity as an independent variable together with an interaction term of Aβ status × Time. Furthermore, we compared the intra-individual variance of a biomarker out of the total variance (IV/TV) in individuals with at least three longitudinal measures. For this, we *z*-scored the biomarkers, calculated intra-individual variance using the deviations from the mean for each participant at every time point, then expressed this as percentage of the total variance for that biomarker. One-tailed significance testing was performed, and confidence intervals were estimated as the bootstrapped *R*^2^ difference [number of iterations (*n*_iter_) = 10 000], restricted to the overlap of participants with all available data during that comparison. The *P*-values were adjusted for multiple comparisons by the Benjamini–Hochberg method (*P* < 0.05 considered significant).

## Results

For the main analyses of cross-sectional fluid biomarker and PET relationships, we used three early-changing Core 1 CSF and plasma AD biomarkers^11^: p-tau217, p-tau181 and Aβ42; two later-changing Core 2 CSF and plasma AD biomarkers^11^: MTBR-tau243 [or endogenously cleaved MTBR-tau243 (eMTBR-tau243)] and p-tau205; two synaptic CSF biomarkers^38^: SNAP-25 and neurogranin; and two CSF and plasma reference proteins: Aβ40 and np-tau. Normalization was performed by calculating the ratio of the fluid biomarker to the reference protein. We included 1702 participants from the BioFINDER-2 cohort [863 (50.7%) male, mean (SD) age 68.4 (12.2) years], all with tau-PET and/or Aβ-PET and at least one plasma or CSF biomarker measure available. For replication, we included 376 and 190 individuals from the Knight ADRC and TRIAD cohorts in the same manner. From the BioFINDER-1 cohort we included 790 participants, all with two or more longitudinal fluid biomarker measures. From the Perugia MS cohort, we included 56 participants, all with a CSF OLINK inflammatory panel and CSF Aβ40 measured. Demographic data for all cohorts are provided in Supplementary Table 1.

### Normalization using CSF Aβ40 enhances CSF AD biomarker associations with tau-PET load

**Figure 1:**
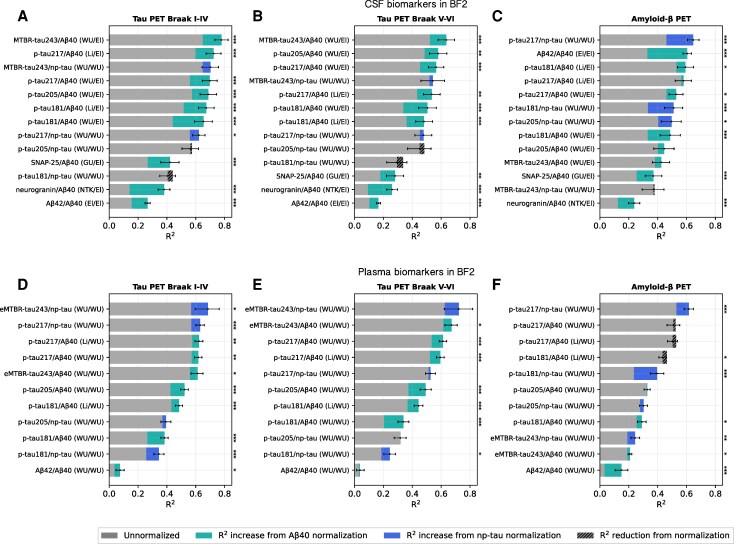
**Reference protein-normalized plasma and CSF biomarkers show stronger associations with tau and Aβ-PET.** The proportion of explained variance (*R*^2^) with and without reference protein normalization for fluid biomarker associations with continuous tau-PET (Braak I–IV and Braak V–VI) or Aβ-PET load in BF2. (**A**–**C**) CSF biomarkers. (**D**–**F**) Plasma biomarkers. Error bars represent 95% confidence intervals for the bootstrapped *R*^2^ difference between the normalized and corresponding unnormalized biomarker. El = Elecsys, immunoassay; GU = University of Gothenburg, mass spectrometry; Li = Lilly, immunoassay; NTK = NeuroToolKit, immunoassay; WU = WU, mass spectrometry. *P < 0.05, **P < 0.01 and ***P < 0.001 compared against the biomarker alone (assessed with bootstrapping and false discover rate corrected).

We compared the proportion of variance (*R*^2^) in tau-PET explained by CSF biomarkers unnormalized or normalized to either CSF Aβ40 or np-tau. Normalization to CSF Aβ40 significantly increased correlations between the CSF biomarkers and tau-PET load in both the temporal meta-ROI and neocortical ROI (Δ*R*^2^ = 0.064–0.24; Figs 1A, B  2A and B, Table 1 and Supplementary Table 2). Normalization to np-tau improved correlations for CSF p-tau217 and CSF MTBR-tau243, but not for CSF p-tau181 or CSF p-tau205.

**Figure 2:**
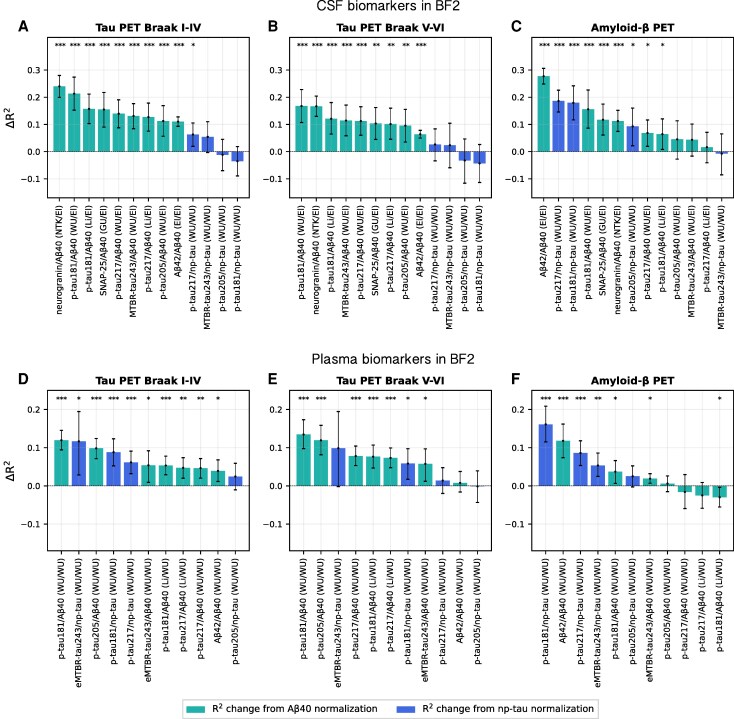
***R* ^2^ change during reference protein normalization for plasma and CSF biomarker associations with tau and Aβ-PET in BF2.** Bar plots showing the difference in proportion of explained variance (Δ*R*^2^) comparing a normalized biomarker with the same biomarker alone in associations with continuous tau-PET (Braak I–IV and Braak V–VI regions of interest) or Aβ-PET load in BF2. (**A**–**C**) CSF biomarkers. (**D**–**F**) Plasma biomarkers. Error bars represent 95% confidence intervals for the bootstrapped *R*^2^ difference between the normalized and unnormalized biomarker. El = Elecsys, immunoassay; GU = University of Gothenburg, mass spectrometry; Li = Lilly, immunoassay; NTK = NeuroToolKit, immunoassay; WU = WU, mass spectrometry. *P < 0.05, **P < 0.01 and ***P < 0.001 compared against the biomarker alone (assessed with bootstrapping and false discovery rate corrected).

**Table 1:** CSF biomarker associations with temporal meta-ROI tau-PET load in BF2

Biomarker	*R* ^2^	Beta	Beta CI lower	Beta CI upper	*n*	Compared against	Δ*R*^2^	*P*-value	*P*-value FDR
MTBRtau243/Aβ40 (WU/El)	0.779	0.882	0.838	0.927	442	MTBRtau243 (WU)	0.131	**<0**.**0001**	**0**.**00021**
MTBRtau243/np-tau (WU/WU)	0.702	0.838	0.787	0.889	442	MTBRtau243 (WU)	0.0559	0.060	0.081
MTBRtau243 (WU)	0.646	0.804	0.748	0.860	442	–	–		–
ptau217/Aβ40 (Li/El)	0.724	0.851	0.814	0.888	774	p-tau217 (Li)	0.127	**<0**.**0001**	**0**.**00021**
p-tau217 (Li)	0.597	0.773	0.728	0.818	774	–	–		–
p-tau217/Aβ40(WU/El)	0.698	0.835	0.784	0.887	442	p-tau217 (WU)	0.139	**<0**.**0001**	**0**.**00021**
p-tau217/np-tau (WU/WU)	0.621	0.788	0.731	0.846	442	p-tau217 (WU)	0.0626	**0**.**0086**	**0**.**013**
p-tau217 (WU)	0.558	0.747	0.684	0.809	442	–	–		–
p-tau181/Aβ40 (Li/El)	0.673	0.821	0.780	0.861	775	p-tau181 (Li)	0.157	**<0**.**0001**	**0**.**00021**
p-tau181 (Li)	0.517	0.719	0.670	0.768	775	–	–		–
p-tau181/Aβ40 (WU/El)	0.654	0.809	0.753	0.864	442	p-tau181 (WU)	0.213	**<0**.**0001**	**0**.**00021**
p-tau181 (WU)	0.441	0.664	0.594	0.734	442	–	–		–
p-tau181/np-tau (WU/WU)	0.404	0.636	0.564	0.708	442	p-tau181 (WU)	−0.036	0.14	0.18
p-tau205/Aβ40 (WU/El)	0.687	0.829	0.776	0.881	442	p-tau205 (WU)	0.112	**0**.**0005**	**0**.**00093**
p-tau205 (WU)	0.572	0.757	0.695	0.818	442	–	–		–
p-tau205/np-tau (WU/WU)	0.562	0.750	0.688	0.812	442	p-tau205 (WU)	−0.0121	0.37	0.38
SNAP-25/Aβ40 (GU/El)	0.422	0.649	0.601	0.697	972	SNAP-25 (UGOT)	0.155	**<0**.**0001**	**0**.**00021**
SNAP-25 (GU)	0.269	0.519	0.465	0.572	972	–	–		**–**
Neurogranin/Aβ40 (NTK/El)	0.3806	0.620	0.569	0.665	1026	Neurogranin (NTK)	0.240	**<0**.**0001**	**0**.**00021**
Neurogranin (NTK)	0.140	0.374	0.317	0.431	1026	–	–		**–**
Aβ42/Aβ40 (El/El)	0.266	−0.516	−0.557	−0.475	1651	Aβ42 (El)	0.110	**<0**.**0001**	**0**.**00021**
Aβ42 (El)	0.156	−0.395	−0.439	−0.351	1651	–	–		–
Aβ40 (El)	0.0001	−0.011	−0.06	0.0368	1659	–	–		–
np-tau181-190 (WU)	0.353	0.594	0.427	0.589	442	–	–		–
np-tau195–210 (WU)	0.407	0.638	0.566	0.710	442	–	–		–
np-tau212–221 (WU)	0.331	0.575	0.498	0.652	442	–	–		–

Univariate linear regression results for CSF biomarkers alone and in ratios with either CSF Aβ40 or non-phosphorylated tau predicting temporal meta-ROI (Braak I–IV) tau PET load. CI = confidence interval; El = Elecsys, immunoassay; FDR = false discovery rate; GU = University of Gothenburg, mass spectrometry; Li = Lilly, immunoassay; NTK = NeuroToolKit, immunoassay; WU = Washington University, mass spectrometry. *P* < 0.05 is denoted in bold.

We next performed replication analyses in the Knight ADRC and TRIAD cohorts. Note that both Knight ADRC and TRIAD had a smaller sample size and smaller proportion of cases with high tau-PET load than BF2, potentially reducing the likelihood of significant findings. Nonetheless, similar effects of increased concordance between tau-PET and CSF biomarkers when normalized to CSF Aβ40 or CSF np-tau, although slightly smaller, were observed, statistically significant for CSF p-tau181, CSF p-tau217, CSF p-tau205, CSF Aβ42, CSF neurogranin and CSF SNAP-25, and trend level for CSF MTBR-tau243 (Supplementary Figs 1A, B, 2A, B, 3A, B, E and F and Supplementary material, Results 1 and 2).

The strongest associations with both temporal meta-ROI (*R*^2^ = 0.78) and neocortical (*R*^2^ = 0.64) tau-PET load in BF2 were achieved with the CSF MTBR-tau243/Aβ40 ratio (in comparison to *R*^2^ = 0.65 and *R*^2^ = 0.52, respectively, for unnormalized CSF MTBR-tau243; Fig. 1A and B, Table 1 and Supplementary Table 2). Likewise, the CSF MTBR-tau243/Aβ40 ratio showed the strongest association with tau-PET in Knight ADRC (Supplementary Fig. 1A and B).

The analyses presented above were done when maximizing the sample size for each biomarker. To perform a head-to-head comparison of all biomarkers, we used a BF2 subset with existing data for all CSF biomarkers and PET outcomes, yielding similar *R*^2^ scores and ranking as in Fig. 1A and B (*n* = 322; Supplementary Fig. 4A and B).

**Figure 3:**
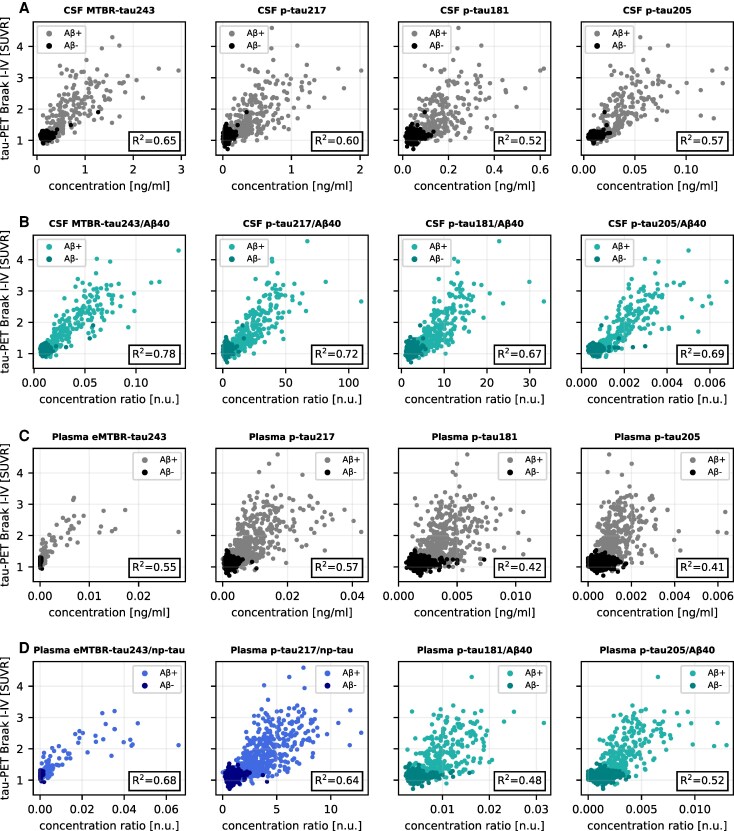
**Scatter plots demonstrating the increased correlation with temporal meta-region of interest tau-PET load for normalized CSF and plasma biomarkers.** Scatter plots of tau-PET Braak I–IV load and fluid biomarkers MTBR-tau243, p-tau217, p-tau181 and p-tau205: (**A**) CSF unnormalized; (**B**) CSF normalized; (**C**) plasma unnormalized; and (**D**) plasma normalized. Mass spectrometry (WU): CSF MTBR-tau243 and p-tau205; plasma eMTBR-tau243, p-tau217 and p-tau205. Immunoassay (Li): CSF p-tau217 and p-tau181; plasma p-tau181.

We next compared normalizing the biomarkers to CSF Aβ42 instead of CSF Aβ40, which for all biomarkers resulted in significantly lower *R*^2^ values (Δ*R*^2^ = 0.061–0.13; Table 2, with a negative Δ*R*^2^ corresponding to CSF Aβ40 normalization performing better than CSF Aβ42 normalization). Scatter plots for all CSF biomarkers and tau-PET in BF2 are shown in Fig. 3A and B and Supplementary Figs 5 and 6.

**Table 2:** Comparison between CSF Aβ42 and CSF Aβ40 normalization

Biomarker	*R* ^2^	Beta	*n*	Compared against	Δ*R*^2^	*P*-value	*P*-value FDR
**Associations with tau PET Braak I–IV**							
p-tau181/Aβ42 (WU/El)	0.523	0.723	442	p-tau181/Aβ40 (WU/El)	−0.125	**<0**.**0001**	**0**.**00023**
p-tau181/Aβ42 (Li/El)	0.547	0.740	775	p-tau181/Aβ40 (Li/El)	−0.119	**<0**.**0001**	**0**.**00023**
p-tau217/Aβ42 (WU/El)	0.561	0.749	442	p-tau217/Aβ40 (WU/El)	−0.131	**<0**.**0001**	**0**.**00023**
p-tau217/Aβ42 (Li/El)	0.590	0.768	774	p-tau217/Aβ40 (Li/El)	−0.125	**<0**.**0001**	**0**.**00023**
MTBR-tau243/Aβ42 (WU/El)	0.642	0.802	442	MTBR-tau243/Aβ40 (WU/El)	−0.131	**<0**.**0001**	**0**.**00023**
p-tau205/Aβ42 (WU/El)	0.586	0.765	442	p-tau205/Aβ40 (WU/El)	−0.0977	**0**.**0034**	**0**.**0051**
Aβ42 (El)	0.156	−0.395	1651	Aβ40 (El)	0.156	**<0**.**0001**	**0**.**00023**
**Associations with tau PET Braak V–VI**							
p-tau181/Aβ42 (WU/El)	0.429	0.655	442	p-tau181/Aβ40 (WU/El)	−0.0798	**0**.**0016**	**0**.**0026**
p-tau181/Aβ42 (Li/El)	0.410	0.640	776	p-tau181/Aβ40 (Li/El)	−0.0696	**0**.**0006**	**0**.**0012**
p-tau217/Aβ42 (WU/El)	0.473	0.688	442	p-tau217/Aβ40 (WU/El)	−0.0838	**0**.**0005**	**0**.**0011**
p-tau217/Aβ42 (Li/El)	0.454	0.674	775	p-tau217/Aβ40 (Li/El)	−0.0755	**0**.**0008**	**0**.**0014**
MTBR-tau243/Aβ42 (WU/El)	0.567	0.753	442	MTBR-tau243/Aβ40 (WU/El)	−0.0919	**<0**.**0001**	**0**.**00023**
p-tau205/Aβ42 (WU/El)	0.500	0.707	442	p-tau205/Aβ40 (WU/El)	−0.0607	**0**.**023**	**0**.**032**
Aβ42 (El)	0.104	−0.322	1652	Aβ40 (El)	0.102	**<0**.**0001**	**0**.**00023**
**Associations with Aβ-PET**							
p-tau181/Aβ42 (WU/El)	0.481	0.693	257	p-tau181/Aβ40 (WU/El)	0.0108	0.43	0.45
p-tau181/Aβ42 (Li/El)	0.623	0.789	507	p-tau181/Aβ40 (Li/El)	0.0344	**0**.**043**	0.056
p-tau217/Aβ42 (WU/El)	0.457	0.676	257	p-tau217/Aβ40 (WU/El)	−0.0664	0.071	0.088
p-tau217/Aβ42 (Li/El)	0.601	0.776	506	p-tau217/Aβ40 (Li/El)	0.0211	0.20	0.23
MTBR-tau243/Aβ42 (WU/El)	0.445	0.667	257	MTBR-tau243/Aβ40 (WU/El)	0.0327	0.23	0.25
p-tau205/Aβ42 (WU/El)	0.461	0.679	257	p-tau205/Aβ40 (WU/El)	0.00849	0.45	0.45
Aβ42 (El)	0.327	−0.572	1145	Aβ40 (El)	0.322	**<0**.**0001**	**0**.**00023**

Univariate linear regression results for CSF biomarkers in ratios with Aβ42 predicting continuous tau and Aβ-PET loads in BF2. This was compared against a ratio with CSF Aβ40. El = Elecsys, immunoassay; FDR = false discovery rate; GU = University of Gothenburg, mass spectrometry; Li = Lilly, immunoassay; NTK = NeuroToolKit, immunoassay; WU = Washington University, mass spectrometry. *P* < 0.05 is denoted in bold.

### Normalization using CSF Aβ40 and np-tau enhances CSF AD biomarker associations with Aβ-PET load

We observed similar results when using continuous Aβ-PET load as outcome: the CSF biomarkers showed stronger associations when normalized to CSF Aβ40 compared with unnormalized (Δ*R*^2^ = 0.016–0.28; Figs 1C and 2C and Supplementary Table 3). Normalization significantly improved *R*^2^ for CSF p-tau181 (Li, WU), CSF p-tau217 (WU), CSF SNAP-25 (UGOT), CSF neurogranin (NTK) and CSF Aβ42 (El), with trend-level increases for CSF p-tau217 (Li), CSF p-tau205 (WU) and CSF MTBR-tau243 (WU) (Fig. 2C and Supplementary Table 3). Normalization to CSF np-tau improved associations with Aβ-PET more than normalization to CSF Aβ40 for CSF p-tau217, CSF p-tau181 and CSF p-tau205 but not CSF MTBR-tau243 in this context. Similar effect sizes and significant increases were seen in the replication cohort Knight ADRC, but less pronounced effects in TRIAD (Supplementary Figs 1C, 2C, 3C and G and Supplementary material, Results 1 and 2).

The strongest Aβ-PET association was observed for CSF p-tau217/np-tau (*R*^2^ = 0.65 versus *R*^2^ = 0.46 for unnormalized CSF p-tau217) in BF2 (Fig. 1C) and in Knight ADRC (Supplementary Fig. 1C). In a head-to-head ranking for all CSF biomarkers in the subset of individuals with all biomarkers available, similar rankings were seen as in Fig. 1C (*n* = 193; Supplementary Fig. 4C). When comparing CSF biomarker normalization using CSF Aβ40 against using CSF Aβ42, no significant differences were seen (Table 2). Scatter plots for all CSF biomarkers and Aβ-PET in BF2 are shown in Supplementary Fig. 7.

### Normalization using plasma Aβ40 and np-tau enhances plasma AD biomarker associations with tau-PET load

We next investigated a similar normalization approach for plasma biomarkers. As for the CSF biomarkers, core plasma AD biomarkers showed significantly higher correlations with tau-PET load in the temporal lobe and neocortex when normalized to plasma Aβ40 or plasma np-tau compared with unnormalized (Δ*R*^2^ = 0.002–0.14; Figs 1D, E, 2D and E and Supplementary Tables 4 and 5). Unlike CSF MTBR-tau243, plasma eMTBR-tau243 showed larger improvement when normalized using plasma np-tau compared with using plasma Aβ40. Both normalization methods, however, yielded a significant increase in *R*^2^ compared with unnormalized plasma eMTBR-tau243. In Knight ADRC, smaller effects but similar trends of improvement were seen when using plasma Aβ40 normalization, but using plasma np-tau did not improve the biomarker associations with tau-PET (Supplementary Figs 1D, E, 2D and E and Supplementary material, Results 1).

The strongest associations with temporal lobe (*R*^2^ = 0.69) and neocortical (*R*^2^ = 0.72) tau load were observed for plasma eMTBR-tau243/np-tau (compared with *R*^2^ = 0.55 and *R*^2^ = 0.60, respectively, for unnormalized plasma eMTBR-tau243; Fig. 1D and E and Supplementary Tables 4 and 5) in BF2.

In a head-to-head biomarker comparison with all data available (*n* = 875; Supplementary Fig. 4D and E), similar *R*^2^ scores and rankings as in Fig. 1D and E were observed for normalized and unnormalized plasma biomarker associations with tau-PET. Note that plasma eMTBR-tau243 was excluded here, owing to plasma samples being from a different visit from the other plasma biomarkers (resulting in no overlap of participants with all data available).

Normalization to plasma Aβ42 instead of plasma Aβ40 yielded significantly higher associations with tau-PET for p-tau181 and eMTBR-tau243, but for the other plasma biomarkers the differences between the two normalization approaches were non-significant (Supplementary Table 6). Note that, in a similar manner to plasma Aβ40, the raw plasma Aβ42 measurements showed very low association with tau-PET (*R*^2^ < 0.037), meaning that plasma Aβ42 might not serve as a disease-related marker in this context (in contrast to CSF Aβ42 in the previous comparisons). Scatter plots for all plasma biomarkers and tau-PET are shown in Fig. 3C and D and Supplementary Figs 8 and 9.

### Normalization using plasma Aβ40 and np-tau enhances plasma AD biomarker associations with Aβ-PET load

For Aβ-PET load, normalization to plasma Aβ40 significantly improved associations for plasma eMTBR-tau243, plasma Aβ42 and plasma p-tau181, whereas plasma np-tau normalization improved plasma eMTBR-tau243, plasma p-tau181 and plasma p-tau217 (Figs 1F and 2F and Supplementary Table 7). Similar effects were seen in Knight ADRC, with significant improvement for plasma Aβ42/Aβ40, plasma p-tau181/np-tau and plasma p-tau217/np-tau, decreased performance for plasma p-tau217/Aβ40 and no difference or trend-level improvement for the other plasma biomarkers (Supplementary Figs 1F and 2F and Supplementary material, Results 1).

The strongest Aβ-PET association was observed for plasma p-tau217/np-tau (*R*^2^ = 0.62 compared with *R*^2^ = 0.53 for unnormalized plasma p-tau217; Fig. 1F and Supplementary Table 7). Similar rankings were seen in a head-to-head comparison between all biomarkers in a subsample with all data available (except plasma eMTBR-tau243, again excluded because samples were measured at a different visit) (*n* = 602; Supplementary Fig. 4F). Normalization using plasma Aβ42 rather than using plasma Aβ40 significantly improved *R*^2^ for plasma p-tau181 and plasma p-tau205, but was non-significant for the other plasma biomarkers (Supplementary Table 6). Again, the raw plasma Aβ42 measurements showed very low association with the outcome Aβ-PET (*R*^2^ = 0.035). Scatter plots for all plasma biomarkers and Aβ-PET are shown in Supplementary Fig. 10.

### Reference protein characteristics and sensitivity analyses

Our previous research has demonstrated that high-performing reference proteins often exhibit low correlations with the dependent variable,^7^ suggesting that an effective reference protein captures sources of variance unrelated to the specific pathology of interest. We observed the same pattern in this study, in that the reference proteins (CSF and plasma Aβ40, Aβ42 and np-tau) improved biomarker associations with tau and Aβ-PET load the most when they individually showed very low correlation with the dependent variable (Tables 1 and 2 and Supplementary Tables 2–7).

For all fluid biomarkers and PET outcomes, three sensitivity analysis were performed in BF2. First, when using Aβ40 or np-tau as a covariate in a multiple linear regression model instead of in a ratio, results generally remained similar (Supplementary material, Results 3). Specifically, adjusting for a reference protein as a covariate typically strengthened associations between fluid biomarkers and tau- or Aβ-PET load, just as ratio normalization did. Notably, Aβ40 and np-tau always had a beta coefficient of opposite sign in comparison to the fluid biomarker, as expected when compensating for interindividual variability. The *R*^2^ rankings of different biomarkers also remained consistent overall. However, using reference proteins as covariates generally resulted in slightly lower *R*^2^ values in comparison to the ratio normalization, for example yielding maximum *R*^2^ values of 0.71 for associations with tau-PET and 0.58 for associations with Aβ-PET, compared with 0.78 and 0.65, respectively, for the ratio approach.

Second, we repeated all analyses in BF2 including only Aβ-positive individuals, to account for the fact that some fluid biomarkers did not exhibit clear linear relationships with the PET outcomes when amyloid-positive and -negative groups were combined (see all scatter plots in Supplementary Figs 5–10). The results remained similar in direction and overall ranking of associations, although the magnitude and statistical significance of some relationships were slightly attenuated, as expected given the reduced sample size and narrower dynamic range (Supplementary material, Results 4).

**Figure 4:**
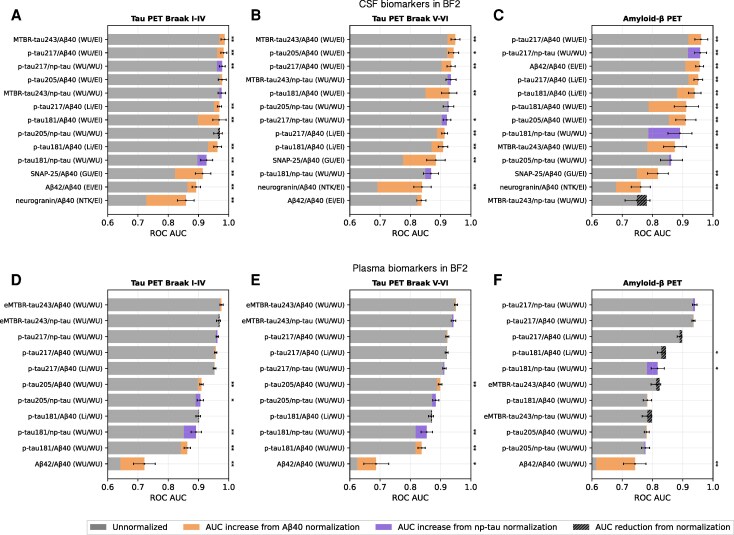
**Reference protein-normalized CSF biomarkers show stronger discrimination between tau and Aβ-positive and -negative individuals.** ROC AUC scores with and without reference protein normalization for fluid biomarker classification of tau and Aβ status in BF2. (**A**–**C**) CSF biomarkers. (**D**–**F**) Plasma biomarkers. Error bars represent 95% confidence intervals for the bootstrapped ROC AUC difference between the normalized and corresponding unnormalized biomarker. El = Elecsys, immunoassay; GU = University of Gothenburg, mass spectrometry; Li = Lilly, immunoassay; NTK = NeuroToolKit, immunoassay; ROC AUC = area under the receiver operating characteristic curve; WU = WU, mass spectrometry. *P < 0.05, **P < 0.01 and ***P < 0.001 compared against the biomarker alone (assessed with bootstrapping and false discovery rate corrected).

Third, we repeated all analyses for Aβ and tau classification instead of regression to provide complementary insights into diagnostic concordances between unnormalized versus normalized fluid biomarkers and PET, without relying on assumptions of linear relationships. For this, we binarized the dependent PET variables using Gaussian mixture modelling, resulting in cut-offs of 1.38 (tau PET Braak I–IV), 1.21 (tau PET Braak V–VI) and 1.02 (Aβ-PET), respectively, closely aligning with recommended cut-offs in previous studies.^39,40^ We then evaluated each fluid biomarker (raw and normalized) in logistic regression models using ROC AUC scores. These analyses demonstrated that reference protein normalization improved classification performance across all CSF biomarkers (Fig. 4), with the highest AUC scores for MTBR-tau243/Aβ40 when differentiating between tau-positive and -negative individuals (AUC = 0.99 for Braak I–IV and AUC = 0.95 for Braak V–VI) and for p-tau217/Aβ40 when differentiating between Aβ-PET-positive and -negative individuals (AUC = 0.96). In contrast, plasma biomarkers generally showed smaller differences between normalized and raw biomarkers, with significant increases in AUC observed only for plasma Aβ42, p-tau181 and p-tau205, whereas the top-performing plasma biomarkers (eMTBR-tau243 and p-tau217) showed no significant increase.

### CSF Aβ40 normalization mainly reduces interindividual variability rather than intra-individual variability over time

**Figure 5:**
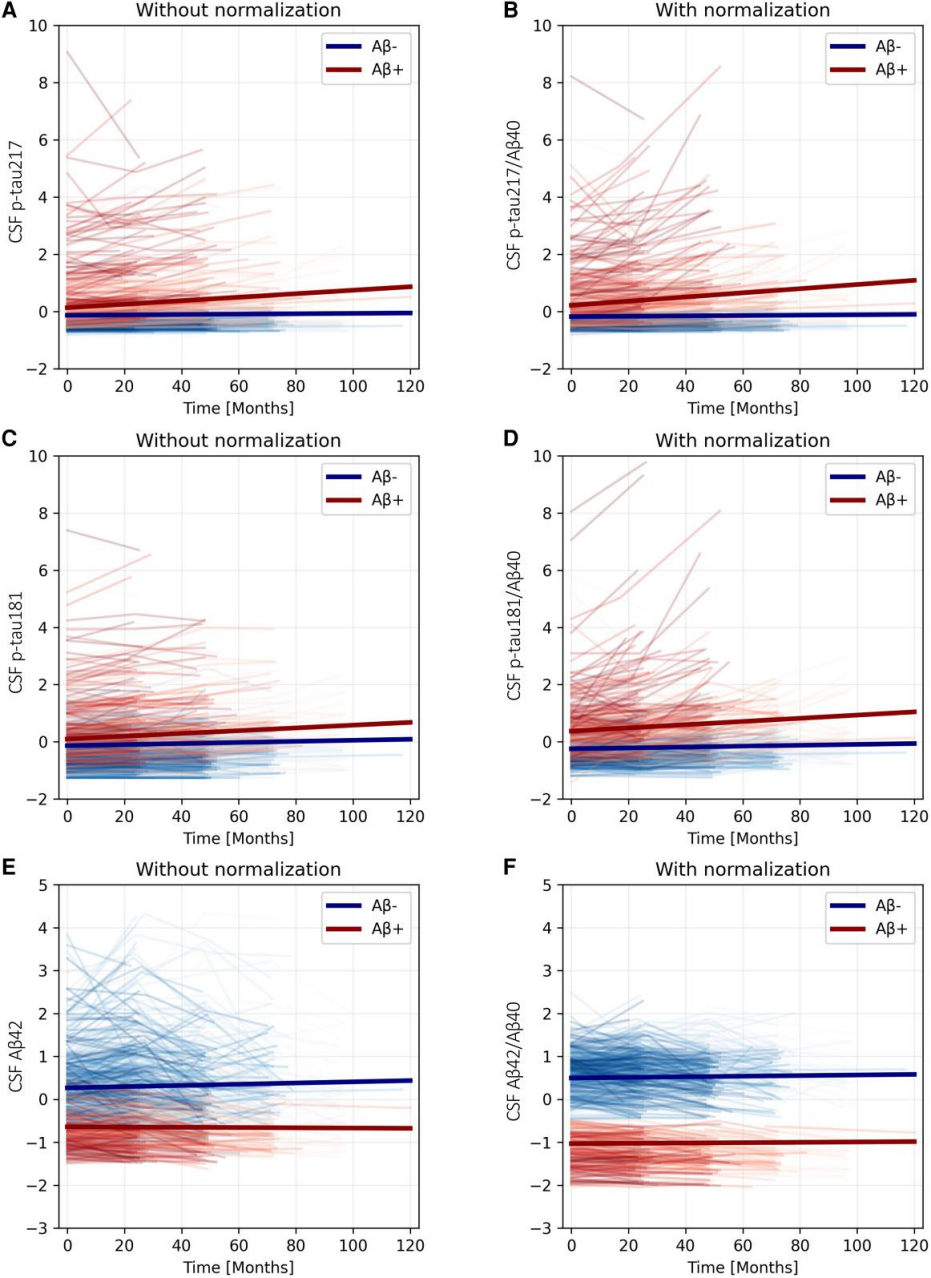
**Normalized CSF biomarkers show a similar change over time to biomarkers alone, but with reduced interindividual variance.** Linear mixed models with independent variables time, Aβ status and Time × Aβ status were fitted to the longitudinal BF1 biomarker data (dependent variable) for CSF: (**A**) p-tau217 (Li); (**C**) p-tau181 (El); and (**E**) Aβ42 (El); and corresponding markers in a ratio with CSF Aβ40 (El) in **B**, **D** and **F**. Further details on all models can be seen in Supplementary Table 9.

For BF1 participants with at least three biennial visits (*n* = 528), we compared the proportion of intra-individual variance of the total variance (IV/TV) for CSF p-tau217, CSF p-tau181 and CSF Aβ42, unnormalized or normalized to CSF Aβ40. The IV/TV generally increased with normalization for both Aβ-positive and -negative individuals (Supplementary Table 8 and Supplementary Fig. 11), indicating that normalization primarily reduced interindividual variability rather than intra-individual variability over time. Further supporting this, for BF1 participants with at least two visits (*n* = 790), linear mixed-effects models showed that CSF Aβ40 normalization reduced participant variance by 39%–80% (estimated from the random intercepts) and increased Aβ-status differentiation, but biomarker changes over time were affected only minimally (Fig. 5 and Supplementary Table 9). Similar trends were observed for plasma p-tau217 and plasma Aβ42, with the largest effect from plasma Aβ40 normalization on variance between participants and Aβ-status differentiation for plasma Aβ42 (Supplementary Fig. 12 and Supplementary Table 10). Comparisons of the longitudinal biomarker changes are provided in Supplementary Fig. 13.

### Exploring CSF Aβ40 normalization for inflammatory CSF markers

We wanted to explore further whether the CSF Aβ40 normalization method translated to other contexts than for core AD CSF biomarkers. We therefore evaluated its effect on group-level differences (Aβ-negative CU versus Aβ-positive AD dementia or MCI) of the microglial CSF sTREM2 and astrocytic CSF YKL-40 biomarkers in BF2. Without normalization, CSF sTREM2 and CSF YKL-40 showed moderate to good discriminative performance, with AUCs of 0.79 and 0.83 for Aβ− CU versus Aβ+ AD dementia, and 0.76 and 0.73 for Aβ− CU versus Aβ+ MCI, respectively. We observed no significant differences in AUC with versus without CSF Aβ40 normalization in this context (ΔAUC < 0.023; Supplementary Fig. 14). For comparison, normalization improved (as expected) several AD biomarkers that initially showed good to excellent discriminative performance (Supplementary Fig. 14): CSF neurogranin (ΔAUCs of ∼0.09 from AUCs of 0.89 and 0.81), CSF SNAP-25 (ΔAUCs = 0.06–0.08 from AUCs of 0.92 and 0.82) and CSF p-tau181 (ΔAUCs = 0.01–0.04 from AUCs of 0.99 and 0.94). We also evaluated associations between CSF sTREM2 and CSF YKL-40 and TSPO-PET (selective for microglial density) in TRIAD with and without CSF Aβ40 normalization, again resulting in no significant differences (Supplementary Fig. 3D and H).

Lastly, to investigate broader translational properties of CSF Aβ40 normalization, we evaluated the method in an MS cohort from the University of Perugia. Note that this cohort was small (*n* = 56), hence the results should be interpreted as preliminary. In the cohort, significantly different CSF proteins from an OLINK CSF inflammatory panel in MS versus other neurological diseases had previously been reported.^22^ We evaluated whether adjustment for CSF Aβ40 levels (which had a low association with all MS outcomes) distinguished these differentiations further, but observed only minor effects in the MS group versus the other neurological diseases group, and in proteins associated with Expanded Disability Status Scale and brain MRI lesions (Supplementary Fig. 15). A larger improvement was generally seen when instead adjusting for a mean CSF level calculated from the proteins with the lowest correlation with the outcome.

## Discussion

In this study, we show that associations between the levels of tau and Aβ pathology in the brain (measured by PET) and several core AD CSF biomarkers are strengthened when the CSF biomarkers are normalized to reference proteins CSF Aβ40 or CSF np-tau. Smaller but similar improvements in association were observed when normalizing core AD plasma biomarkers to reference proteins plasma Aβ40 or plasma np-tau. Improvements when using a reference protein were seen for multiple AD biomarkers, mass spectrometry-based assays (WU and GU) and immunoassays (Li, El and NTK), applying the reference protein as a ratio denominator and a covariate, and in three independent cohorts (BF2, Knight ADRC and TRIAD), underscoring the robustness of the method. These findings can improve the precision and utility of fluid biomarkers in AD, highly relevant for both scientific and clinical applications.

Until now, there has been a lack of extensive studies comparing different fluid biomarker normalization methods in relationship to AD pathology. Although CSF Aβ40 has previously been suggested as a potential reference protein for CSF Aβ42 and CSF p-tau181 by us and others,^1,7,8,10,41^ our results suggest that such normalization might also benefit many more core CSF AD biomarkers (e.g. CSF MTBR-tau243, CSF p-tau217 and CSF p-tau205). Likewise, although normalization of plasma biomarkers has been shown to be beneficial in the context of plasma Aβ42/Aβ40 and plasma p-tau/np-tau (%p-tau),^28,42-44^ our study further highlights the benefit of using reference proteins to normalize additional core AD plasma biomarkers (e.g. by normalizing plasma eMTBR-tau243, plasma p-tau217, plasma p-tau181 and plasma p-tau205 using plasma Aβ40 for increased associations with tau-PET). The result that reference protein normalization improves concordance between fluid biomarkers and PET is important in the context of the recently proposed biological diagnosis and staging for AD,^11^ and is particularly relevant for when imaging, CSF and blood biomarkers are intended to be used interchangeably.

We observed that normalization to a non-disease-related marker, such as CSF Aβ40, generally outperformed the use of another disease-related biomarker (e.g. CSF Aβ42) as the ratio denominator. Furthermore, CSF np-tau, plasma np-tau and plasma Aβ42 performed best as reference proteins when they had relatively low correlation with the dependent variable. This suggests that an optimal reference protein should capture variance related to general proteomic or biofluid characteristics rather than disease-specific alterations. Interestingly, however, CSF Aβ40 normalization did not enhance performance of inflammatory CSF markers in AD or MS, despite its weak association with the dependent variables alone. In our previous work, we observed that CSF Aβ40 worked well as a reference protein, but other candidate proteins were more strongly correlated with the average CSF protein abundance level, potentially limiting the ability of CSF Aβ40 to represent individual proteomic variability in a general manner.^7^ Another hypothesis is that an optimal reference protein should be more closely related to the biological pathway of the biomarker of interest, because neuronally derived biomarkers (e.g. CSF MTBR-tau243, CSF p-tau217, CSF neurogranin and CSF SNAP-25) benefitted from CSF Aβ40 normalization, whereas neuroinflammatory markers (e.g. CSF sTREM2 and CSF YKL-40) might be more enhanced by reference proteins secreted by microglia or astrocytes (we observed, for example, that a mean of several inflammatory markers improved the biomarkers slightly more than CSF Aβ40 normalization in the MS cohort). In line with this hypothesis and extending beyond core AD biomarkers, Nilsson *et al*.^38^ and Oh *et al*.^45^ previously showed that ratios of synaptic CSF proteins (e.g. CSF YWHAG/NPTX2 and CSF SNAP-25/NPTX) were strong predictors of cognitive impairment. Likewise, Mravinacová *et al*.^46^ previously concluded that combining brain-derived proteins in pairs (e.g. in a ratio) increased their correlation with cognitive decline. Given the small sample size in the MS cohort, our analysis on inflammatory biomarkers was limited, and we encourage future studies to continue investigating reference proteins for contexts beyond AD biomarkers.

The consistent increase in associations between fluid biomarkers and PET with reference protein normalization across cohorts and PET tracers underscores the methodological robustness of this approach. Although such associations are clinically relevant (given that fluid and imaging biomarkers often are used interchangeably), the assumptions of linear relationships between fluid and imaging biomarkers have limitations from biological and technical perspectives. For example, fluid and PET biomarkers often follow non-linear trajectories owing to plateau or saturation effects, and different biomarkers reflect distinct temporal phases of amyloid and tau pathology.^20,47,48^ This is likely to prohibit fluid and PET biomarkers from reaching perfect linear correlations. In the present study, we acknowledged the non-linear relationships by visualizing scatter plots, analysing associations separately within the Aβ-positive subgroup, and demonstrating the benefits of reference protein normalization using classification approaches that rely on binarization of outcomes rather than assuming linearity across the entire dynamic range. Apart from non-linear relationships, technical factors, such as tracer binding properties and cohort composition (e.g. distribution of disease stages), can also influence biomarker specificity, sensitivity and dynamic range, thereby affecting interpretations. We addressed this by showing that reference protein normalization consistently improved biomarkers across several PET tracers and AD cohorts with different disease stage distributions. Careful consideration of factors like these will be essential to interpret biomarker relationships appropriately across different research and clinical contexts also in future studies.

Although Aβ40 worked well as a reference protein in both CSF and plasma, we acknowledge that CSF and plasma properties might not always be directly transferable, because the two biofluids are involved in different physiological processes and exhibit notable compositional differences. CSF Aβ40 is, for example, produced predominantly within the CNS, whereas plasma Aβ40 originates largely from the peripheral nervous system.^49^ Additionally, plasma Aβ40 levels, but not CSF Aβ40 levels, have been found to be highly affected by oral intake of neprilysin inhibitors.^50^ Future studies are needed to gain a better understanding of the fluid properties that reference proteins represent in plasma.

Longitudinal analyses revealed that the fluid biomarker ratios reduced interindividual variability to a larger extent than intra-individual variability over time, as shown by IV/TV analysis and linear mixed-effects models. Previously, we and others have shown that interindividual CSF proteomic variability is related to age, sex and ventricular volume,^7,51-53^ which are relatively constant intra-individual factors. Although reference protein normalization might address these differences, a ratio can be more sensitive to measurement errors because two errors are present instead of one, which might particularly impact markers that are otherwise stable within individuals over time. Thus, our findings further support that reference protein normalization reduces interindividual variability, whereas its added value for minimizing intra-individual fluctuations over time might be more limited. Nonetheless, given that ventricular volume and related factors explain only a modest proportion of the total biomarker variance in healthy individuals,^7,51,53^ additional studies are needed to clarify the role of reference proteins for longitudinal monitoring, which is important when tracking drug-induced effects on fluid biomarkers in clinical practice and trials.

### Limitations

Key limitations include varying sample availability across analyses and the small Perugia MS cohort. We included all available data for each analysis to maximize statistical power, followed by significance tests and subsample analyses with datasets with all data available. We also focused only on linear relationships, which might not capture more complex biomarker interactions. Additionally, demographic biases (e.g. predominantly highly educated, white participants) warrant further validation in more diverse populations, despite the demonstrated generalizability of the biomarker normalization method across biomarkers, assays and cohorts.

## Conclusion

Normalizing core CSF AD biomarkers to reference proteins CSF Aβ40 or CSF np-tau, and plasma AD biomarkers to reference proteins plasma Aβ40 or plasma np-tau, significantly improved their associations with tau- and Aβ-PET. These improvements were seen for multiple AD biomarkers, using both mass spectrometry-based assays and immunoassays, when applying the reference protein as a ratio denominator or as a covariate, and across three independent AD cohorts, underscoring the robustness and generalizability of the method. The findings can enhance the precision and utility of AD fluid biomarkers, with important implications for both research and clinical applications, and particularly relevant for when imaging, CSF and blood biomarkers are intended to be used interchangeably.

## Supplementary Material

awaf375_Supplementary_Data

## Data Availability

The datasets generated and/or analysed during the present study are available from the Principal Investigators of the respective cohort (bf_executive@med.lu.se for the Swedish BioFINDER-1 and BioFINDER-2, R.J.B. for Knight ADRC, P.R.-N. for TRIAD, and L.P. for the Perugia MS cohort). Generally, anonymized data can be shared by request from qualified academic investigators for the purpose of replicating procedures and results presented in the article, if data transfer is in agreement with data protection regulation at the institution and is approved by the local ethics review board.
