# Supplementary material for: Reference proteins to improve Core 1 and Core 2 Alzheimer’s disease CSF and plasma biomarkers
Source: Brain. 2025 Oct 6;149(4):1153–67. doi: 10.1093/brain/awaf375 (PMC13058454; doi:10.1093/brain/awaf375)
Supplement: awaf375_Supplementary_Data [file awaf375_supplementary_data.zip › brain-2025-01206-File009.pdf]

Supplementary Table 1: Cohort demographics. Abbreviations: Mini Mental State Examination (MMSE), positron emission tomography (PET), normal cognition (NC), subjective cognitive decline (SCD), mild cognitive impairment (MCI), standardized uptake value ratio (SUVR), other neurological disease (OND), multiple sclerosis (MS), expanded disability status scale (EDSS), gadolinium magnetic resonance imaging (GD MRI), *missing data for some participants

	**BioFINDER-2**	**BioFINDER-1**	**KnightADRC**	**TRIAD**	**Perugia MS**
**n**	1702	790	376	190	56
**Age [years]**	68.4 (12.2)	72.2 (5.31)	72.2 (6.27)	61.4 (17.8)	43.8 (14.6)
**Sex male (%)**	863 (50.7%)	399 (50.5%)	160 (42.6%)	84 (44.2%)	15 (27%)
**Education [years]**	12.7 (3.82)	12.1 (3.68)	16.3 (2.47)	15.1 (3.28)	-
**MMSE**	26.8 (3.85)	28.3 (1.69)	29.1 (1.64)	27.6 (4.05)	-
**EDSS**	-	-	-	-	2.15 (1.40)
***APOE* ε4 carrier* (%)**	776/1562 (49.7%)	299/785 (38.1%)	81/241 (33.6%)	70 (36.8%)	-
**Clinical Diagnosis***					
NC	637	462	286	116	-
SCD	257	142	-	-	-
MCI	406	172	-	35	-
Dementia	390	14	49	37	-
OND/TBD	4	-	-	2	14
MS	-	-	-	-	42
**Aβ-positive* (%)**	769/1692 (45.4%)	272/772 (35.2%)	131/334 (39.2%)	69/188 (36.7%)	-
**Tau-PET**					
n	1702	-	337	189	-
Braak I-IV [SUVR]	1.35 (0.465)	-	1.20 (0.193)	1.21 (0.623)	-
Braak V-VI [SUVR]	1.14 (0.262)	-	1.08 (0.112)	1.24 (0.544)	-
**Aβ-PET**					
n	1173	-	447	188	-
Neocortical composite	1.11 (0.302) [SUVR]	-	22.5 (34.9) [Centiloids]	1.62 (0.548) [SUVR]	-
**TSPO-PET**					
n	-	-	-	81	-
Posterior cingulate cortex [SUVR]	-	-	-	1.09 (0.888)	-
**≥ 1 GD MRI lesions (only in MS)**	-	-	-	-	19/42
**# Visits (every two years)**					
2	-	790	-	-	-
3	-	521	-	-	-
4	-	223	-	-	-
5	-	18	-	-	-

Supplementary Table 2: CSF biomarker associations with neocortical tau-PET load in BF2. Univariate linear regression results for CSF biomarkers alone and in ratios with either Aβ or non-phosphorylated tau predicting continuous neocortical (Braak V-VI) tau PET load. The biomarkers tested included both mass spectrometry-based assays (WU and GU) and immunoassays (Li, NTK and El). Turquoise represents a ratio with Aβ40, blue with np-tau and black the biomarker alone. One-tailed significance testing was performed as bootstrapped R^2 difference (n_{iter}=10,000), restricted to the overlap of participants with all available data during that comparison. A P-value less than 0.05 indicated statistical significance (denoted in bold), and P-values were adjusted for multiple comparisons by the Benjamini–Hochberg method.

Biomarker	R^2	Beta	Beta conf int lower	Beta conf int upper	N	Compared against	ΔR^2	P-value	P-value FDR corrected
**MTBR-tau243/Aβ40 (WU/El)**	0.636	0.797	0.741	0.854	442	MTBR-tau243 (WU)	0.114	**<0.0001**	**0.00021**
**MTBR-tau243/np-tau (WU/WU)**	0.542	0.736	0.672	0.799	442	MTBR-tau243 (WU)	0.0241	0.32	0.33
MTBR-tau243 (WU)	0.520	0.721	0.656	0.786	442				
**p-tau205/Aβ40 (WU/El)**	0.580	0.761	0.701	0.822	442	p-tau205 (WU)	0.0947	**0.0050**	**0.0081**
p-tau205 (WU)	0.484	0.695	0.628	0.763	442				
**p-tau205/np-tau (WU/WU)**	0.450	0.671	0.602	0.741	442	p-tau205 (WU)	-0.0325	0.26	0.29
**p-tau217/Aβ40 (WU/El)**	0.565	0.752	0.690	0.814	442	p-tau217 (WU)	0.113	**<0.0001**	**0.00021**
**p-tau217/np-tau (WU/WU)**	0.478	0.691	0.623	0.759	442	p-tau217 (WU)	0.0259	0.23	0.26
p-tau217 (WU)	0.452	0.673	0.603	0.742	442				
**p-tau217/Aβ40 (Li/El)**	0.535	0.731	0.683	0.780	775	p-tau217 (Li)	0.101	**0.0007**	**0.0012**
p-tau217 (Li)	0.433	0.658	0.605	0.711	775				
**p-tau181/Aβ40 (WU/El)**	0.506	0.711	0.645	0.777	442	p-tau181 (WU)	0.168	**<0.0001**	**0.00021**
p-tau181 (WU)	0.338	0.581	0.505	0.657	442				
**p-tau181/np-tau (WU/WU)**	0.292	0.541	0.462	0.619	442	p-tau181 (WU)	-0.0441	0.16	0.19
**p-tau181/Aβ40 (Li/El)**	0.481	0.693	0.643	0.744	776	p-tau181 (Li)	0.121	**<0.0001**	**0.00021**
p-tau181 (Li)	0.360	0.600	0.543	0.656	776				

**SNAP-25/Aβ40 (GU/EI)**	0.281	0.530	0.477	0.583	973	SNAP-25 (UGOT)	0.103	**0.0018**	**0.0031**
SNAP-25 (GU)	0.180	0.424	0.367	0.481	973				
**Neurogranin/Aβ40 (NTK/EI)**	0.259	0.509	0.457	0.562	1027	Neurogranin (NTK)	0.167	**<0.0001**	**0.00021**
Neurogranin (NTK)	0.0917	0.303	0.245	0.361	1027				
**Aβ42/Aβ40 (EI/EI)**	0.165	-0.407	-0.451	-0.363	1652	Aβ42 (EI)	0.0639	**<0.0001**	**0.00021**
Aβ42 (EI)	0.102	-0.319	-0.365	-0.273	1652				
Aβ40 (EI)	0.0009	-0.0300	-0.078	0.018	1660				
np-tau181-190 (WU)	0.275	0.524	0.445	0.604	442				
np-tau195-210 (WU)	0.330	0.575	0.498	0.651	442				
np-tau212-221 (WU)	0.258	0.508	0.427	0.589	442				

Supplementary Table 3: CSF biomarker associations with Aβ-PET in BF2. Univariate linear regression results for CSF biomarkers alone and in ratios with either Aβ40 or non-phosphorylated tau predicting continuous cortical Aβ-PET load. The biomarkers tested included both mass spectrometry-based assays (WU and GU) and immunoassays (Li, NTK and El). Turquoise represents a ratio with Aβ40, blue with np-tau and black the biomarker alone. One-tailed significance testing was performed as bootstrapped R^2 difference (n_{iter}=10,000), restricted to the overlap of participants with all available data during that comparison. A P-value less than 0.05 indicated statistical significance (denoted in bold), and P-values were adjusted for multiple comparisons by the Benjamini–Hochberg method.

Biomarker	R^2	Beta	Beta conf int lower	Beta conf int upper	n	Compared against	ΔR^2	P-value	P-value FDR corrected
**p-tau217/np-tau (WU/WU)**	0.652	0.807	0.734	0.880	257	p-tau217 (WU)	0.188	**<0.0001**	**0.00021**
**p-tau217/Aβ40 (WU/El)**	0.528	0.727	0.642	0.812	257	p-tau217 (WU)	0.0688	**0.010**	**0.015**
p-tau217 (WU)	0.460	0.678	0.588	0.769	257				
**Aβ42/Aβ40 (El/El)**	0.605	0.778	0.814	0.742	1145	Aβ42 (El)	0.278	**<0.0001**	**0.00021**
Aβ42 (El)	0.328	0.573	0.620	0.525	1145				
**p-tau181/Aβ40 (Li/El)**	0.593	0.770	0.714	0.826	507	p-tau181 (Li)	0.0639	**0.030**	**0.042**
p-tau181 (Li)	0.530	0.728	0.668	0.788	507				
**p-tau217/Aβ40 (Li/El)**	0.580	0.761	0.705	0.818	506	p-tau217 (Li)	0.0161	0.31	0.33
p-tau217 (Li)	0.564	0.751	0.694	0.809	506				
**p-tau181/np-tau (WU/WU)**	0.513	0.716	0.630	0.802	257	p-tau181 (WU)	0.180	**<0.0001**	**0.00021**
**p-tau181/Aβ40 (WU/El)**	0.487	0.698	0.609	0.786	257	p-tau181 (WU)	0.155	**<0.0001**	**0.00021**
p-tau181 (WU)	0.332	0.576	0.476	0.677	257				
**p-tau205/np-tau (WU/WU)**	0.498	0.706	0.619	0.793	257	p-tau205 (WU)	0.0928	**0.019**	**0.027**
**p-tau205/Aβ40 (WU/El)**	0.446	0.668	0.576	0.759	257	p-tau205 (WU)	0.0468	0.15	0.18
p-tau205 (WU)	0.399	0.632	0.536	0.728	257				

**MTBR-tau243/Aβ40 (WU/EI)**	0.424	0.652	0.558	0.745	257	MTBR-tau243 (WU)	0.0437	0.12	0.15
MTBR-tau243 (WU)	0.381	0.617	0.520	0.714	257				
**MTBR-tau243/np-tau (WU/WU)**	0.374	0.611	0.514	0.709	257	MTBR-tau243 (WU)	-0.0059	0.45	0.45
**SNAP-25/Aβ40 (GU/EI)**	0.371	0.609	0.548	0.670	646	SNAP-25 (UGOT)	0.117	**0.0004**	**0.00078**
SNAP-25 (GU)	0.253	0.503	0.436	0.570	646				
**Neurogranin/Aβ40 (NTK/EI)**	0.237	0.487	0.421	0.552	682	Neurogranin (NTK)	0.113	**<0.0001**	**0.00021**
Neurogranin (NTK)	0.124	0.352	0.282	0.423	682				
Aβ40 (EI)	0.004	0.0632	0.0054	0.121	1151				
np-tau181-190 (WU)	0.196	0.443	0.333	0.555	257				
np-tau195-210 (WU)	0.253	0.503	0.396	0.610	257				
np-tau212-221 (WU)	0.182	0.426	0.315	0.538	257				

Supplementary Table 4: Plasma biomarker associations with temporal meta-ROI tau-PET load in BF2. Univariate linear regression results for plasma biomarkers alone and in ratios with either Aβ40, Aβ42 or non-phosphorylated tau predicting continuous temporal meta-ROI (Braak I-IV) tau PET load. The biomarkers tested included both mass spectrometry-based assays (WU) and immunoassays (Li). Turquoise represents a ratio with Aβ40, blue with np-tau and black the biomarker alone. One-tailed significance testing was performed as bootstrapped R^2 difference (n_{iter}=10,000), restricted to the overlap of participants with all available data during that comparison. A P-value less than 0.05 indicated statistical significance (denoted in bold), and P-values were adjusted for multiple comparisons by the Benjamini–Hochberg method.

Biomarker	R^2	Beta	Beta conf int lower	Beta conf int upper	n	Compared against	ΔR^2	P-value	P-value FDR corrected
**eMTBR-tau243/np-tau** (WU/WU)	0.685	0.827	0.739	0.915	161	eMTBR-tau243 (WU)	0.117	**0.012**	**0.017**
**eMTBR-tau243/Aβ40** (WU/WU)	0.612	0.783	0.685	0.880	161	eMTBR-tau243 (WU)	0.0539	**0.022**	**0.026**
eMTBR-tau243 (WU)	0.549	0.741	0.635	0.846	161				
**p-tau217/np-tau** (WU/WU)	0.644	0.803	0.765	0.840	982	p-tau217 (WU)	0.0724	**0.0003**	**0.00062**
**p-tau217/Aβ40** (WU/WU)	0.628	0.793	0.754	0.831	982	p-tau217 (WU)	0.0573	**0.0015**	**0.0029**
p-tau217 (WU)	0.570	0.755	0.714	0.796	982				
**p-tau217/Aβ40** (Li/WU)	0.626	0.792	0.751	0.832	889	p-tau217 (Li)	0.0544	**0.0017**	**0.0031**
p-tau217 (Li)	0.571	0.756	0.713	0.799	889				
**p-tau205/Aβ40** (WU/WU)	0.520	0.721	0.677	0.766	945	p-tau205 (WU)	0.102	**<0.0001**	**0.00025**
**p-tau205/np-tau** (WU/WU)	0.480	0.693	0.646	0.739	945	p-tau205 (WU)	0.0610	**0.0192**	**0.030**
p-tau205 (WU)	0.412	0.645	0.596	0.694	945				
**p-tau181/Aβ40** (Li/WU)	0.481	0.694	0.646	0.741	889	p-tau181 Li	0.0569	**0.0002**	**0.00045**
p-tau181 (Li)	0.424	0.651	0.601	0.701	889				
**p-tau181/Aβ40** (WU/WU)	0.377	0.614	0.565	0.664	983	p-tau181 (WU)	0.120	**<0.0001**	**0.00025**
**p-tau181/np-tau** (WU/WU)	0.343	0.585	0.534	0.636	983	p-tau181 (WU)	0.0844	**<0.0001**	**0.00025**
p-tau181 (WU)	0.256	0.506	0.451	0.560	983				
**Aβ42/Aβ40** (WU/WU)	0.0807	-0.284	-0.344	-0.224	987	Aβ42 (WU)	0.0443	**0.008**	**0.014**

Aβ42 (WU)	0.0366	-0.191	-0.253	-0.130	987				
Aβ40 (WU)	1.89e-5	-0.0043	-0.0669	0.0582	987				
np-tau181-190 (WU)	0.0849	0.291	0.231	0.351	983				
np-tau195-210 (WU)	0.130	0.361	0.301	0.420	945				
np-tau212-221 (WU)	0.0428	0.207	0.146	0.268	982				

Supplementary Table 5: Plasma biomarker associations with neocortical tau PET load in BF2. Univariate linear regression results for plasma biomarkers alone and in ratios with either Aβ40, Aβ42 or non-phosphorylated tau predicting continuous neocortical (Braak V-VI) tau PET load. The biomarkers tested included both mass spectrometry-based assays (WU) and immunoassays (Li). Turquoise represents a ratio with Aβ40, blue with np-tau and black the biomarker alone. One-tailed significance testing was performed as bootstrapped R^2 difference (n_{iter}=10,000), restricted to the overlap of participants with all available data during that comparison. A P-value less than 0.05 indicated statistical significance (denoted in bold), and P-values were adjusted for multiple comparisons by the Benjamini–Hochberg method.

Biomarker	R^2	Beta	Beta conf int lower	Beta conf int upper	n	Compared against	ΔR^2	P-value	P-value FDR corrected
**eMTBR-tau243/np-tau** (WU/WU)	0.723	0.850	0.768	0.933	161	eMTBR-tau243 (WU)	0.0987	0.056	0.056
**eMTBR-tau243/Aβ40** (WU/WU)	0.672	0.820	0.723	0.909	161	eMTBR-tau243 (WU)	0.0576	**0.0051**	**0.015**
eMTBR-tau243 (WU)	0.601	0.775	0.676	0.874	161				
**p-tau217/Aβ40** (WU/WU)	0.612	0.782	0.743	0.821	982	p-tau217 (WU)	0.0786	**<0.0001**	**0.00025**
**p-tau217/np-tau** (WU/WU)	0.568	0.754	0.712	0.795	982	p-tau217 (WU)	0.0356	0.08	0.11
p-tau217 (WU)	0.532	0.729	0.686	0.772	982				
**p-tau217/Aβ40** (Li/WU)	0.594	0.771	0.729	0.813	889	p-tau217 (Li)	0.0730	**<0.0001**	**0.00025**
p-tau217 (Li)	0.519	0.721	0.675	0.766	889				
**p-tau205/Aβ40** (WU/WU)	0.492	0.701	0.656	0.747	945	p-tau205 (WU)	0.120	**<0.0001**	**0.00025**
**p-tau205/np-tau** (WU/WU)	0.370	0.609	0.556	0.656	945	p-tau205 (WU)	0.00193	0.48	0.49
p-tau205 (WU)	0.369	0.607	0.556	0.658	945				
**p-tau181/Aβ40** (Li/WU)	0.443	0.666	0.617	0.715	889	p-tau181 (Li)	0.0768	**<0.0001**	**0.00025**
p-tau181 (Li)	0.365	0.604	0.552	0.657	889				
**p-tau181/Aβ40** (WU/WU)	0.338	0.582	0.531	0.633	983	p-tau181 (WU)	0.135	**<0.0001**	**0.00025**
**p-tau181/np-tau** (WU/WU)	0.261	0.511	0.457	0.565	983	p-tau181 (WU)	0.0577	**0.013**	**0.020**
p-tau181 (WU)	0.199	0.447	0.390	0.503	983				
**Aβ42/Aβ40** (WU/WU)	0.0337	-0.184	-0.245	-0.122	987	Aβ42 (WU)	0.00393	0.36	0.37

Aβ42 (WU)	0.0327	-0.181	-0.242	-0.119	987				
Aβ40 (WU)	0.0027	-0.052	-0.115	0.010	987				
np-tau181-190 (WU)	0.0638	0.253	0.192	0.313	983				
np-tau195-210 (WU)	0.124	0.352	0.292	0.411	945				
np-tau212-221 (WU)	0.0381	0.195	0.134	0.257	982				

Supplementary Table 6: Comparison between plasma Aβ42 and Aβ40 as denominator in biomarker ratios. Univariate linear regression results for plasma biomarkers in ratios with Aβ42 predicting continuous tau and Aβ-PET loads in BF2. This was compared against a ratio with plasma Aβ40. A ratio with plasma Aβ42 yielded significantly higher R^2 for p-tau181 and all PET outcomes, otherwise differences were non-significant. Turquoise represents a ratio with Aβ40, orange a ratio with Aβ42 and black the biomarker alone.

Biomarker	R^2	Beta	n	Compared against	ΔR^2	P-value	P-value FDR corrected
**Associations with tau PET Braak I-IV**							
p-tau181/Aβ42 (WU/WU)	0.409	0.639	983	p-tau181/Aβ40 (WU/WU)	0.0317	**0.0029**	**0.0087**
p-tau181/Aβ42 (Li/WU)	0.506	0.711	889	p-tau181/Aβ40 (Li/WU)	0.0241	**0.0036**	**0.0093**
p-tau217/Aβ42 (WU/WU)	0.621	0.788	982	p-tau217/Aβ40 (WU/WU)	-0.00643	0.26	0.28
p-tau217/Aβ42 (Li/WU)	0.639	0.800	889	p-tau217/Aβ40 (Li/WU)	0.0128	0.073	0.12
eMTBR-tau243/Aβ42 (WU/WU)	0.594	0.771	162	eMTBR-tau243/Aβ40 (WU/WU)	0.0582	**0.0050**	**0.011**
p-tau205/Aβ42 (WU/WU)	0.532	0.730	945	p-tau205/Aβ40 (WU/WU)	0.0129	0.12	0.16
Aβ42 (WU)	0.0390	-0.198	987	Aβ40 (WU)	0.0388	**0.0019**	**0.0068**
**Associations with tau PET Braak V-VI**							
p-tau181/Aβ42 (WU/WU)	0.336	0.580	983	p-tau181/Aβ40 (WU/WU)	0.0235	**0.019**	**0.043**
p-tau181/Aβ42 (Li/WU)	0.431	0.656	889	p-tau181/Aβ40 (Li/WU)	0.0163	**0.044**	0.088
p-tau217/Aβ42 (WU/WU)	0.577	0.760	982	p-tau217/Aβ40 (WU/WU)	-0.00612	0.27	0.28
p-tau217/Aβ42 (Li/WU)	0.578	0.760	889	p-tau217/Aβ40 (Li/WU)	0.00789	0.17	0.21
eMTBR-tau243/Aβ42 (WU/WU)	0.652	0.808	162	eMTBR-tau243/Aβ40 (WU/WU)	0.0100	0.26	0.27
p-tau205/Aβ42 (WU/WU)	0.476	0.690	945	p-tau205/Aβ40 (WU/WU)	0.0100	0.19	0.23
Aβ42 (WU)	0.0409	-0.202	987	Aβ40 (WU)	0.0390	**<0.0001**	**0.00045**
**Associations with Aβ-PET**							
p-tau181/Aβ42 (WU/WU)	0.381	0.617	685	p-tau181/Aβ40 (WU/WU)	0.0927	**<0.0001**	**0.00045**
p-tau181/Aβ42 (Li/WU)	0.513	0.717	612	p-tau181/Aβ40 (Li/WU)	0.0670	**<0.0001**	**0.00045**
p-tau217/Aβ42 (WU/WU)	0.575	0.758	683	p-tau217/Aβ40 (WU/WU)	-0.00741	0.30	0.30
p-tau217/Aβ42 (Li/WU)	0.562	0.750	612	p-tau217/Aβ40 (Li/WU)	0.0249	0.055	0.098
eMTBR-tau243/Aβ42 (WU/WU)	0.195	0.442	109	eMTBR-tau243/Aβ40 (WU/WU)	0.0100	0.12	0.16
p-tau205/Aβ42 (WU/WU)	0.380	0.616	653	p-tau205/Aβ40 (WU/WU)	0.0462	**<0.0001**	**0.00045**
Aβ42 (WU)	0.0348	-0.187	687	Aβ40 (WU)	0.0231	0.096	0.14

Supplementary Table 7: Plasma biomarker associations with Aβ-PET in BF2. Univariate linear regression results for plasma biomarkers alone and in ratios with either Aβ40, Aβ42 or non-phosphorylated tau predicting continuous cortical Aβ-PET load. The biomarkers tested included both mass spectrometry-based assays (WU) and immunoassays (Li). Turquoise represents a ratio with Aβ40, blue with np-tau and black the biomarker alone. One-tailed significance testing was performed as bootstrapped R^2 difference (n_{iter}=10,000), restricted to the overlap of participants with all available data during that comparison. A P-value less than 0.05 indicated statistical significance (denoted in bold), and P-values were adjusted for multiple comparisons by the Benjamini–Hochberg method.

Biomarker	R^2	Beta	Beta conf int lower	Beta conf int upper	n	Compared against	ΔR^2	P-value	P-value FDR corrected
**p-tau217/np-tau (WU/WU)**	0.621	0.788	0.741	0.834	683	p-tau217 (WU)	0.08403	**<0.0001**	**0.00025**
p-tau217 (WU)	0.532	0.730	0.678	0.781	683				
**p-tau217/Aβ40 (WU/WU)**	0.508	0.713	0.660	0.766	683	p-tau217 (WU)	-0.0154	0.36	0.37
p-tau217 (Li)	0.530	0.728	0.674	0.783	612				
**p-tau217/Aβ40 (Li/WU)**	0.503	0.709	0.653	0.765	612	p-tau217 (Li)	-0.0246	0.13	0.16
p-tau181 (Li)	0.465	0.682	0.624	0.740	612				
**p-tau181/Aβ40 (Li/WU)**	0.434	0.659	0.599	0.718	612	p-tau181 (Li)	-0.0296	**0.028**	**0.040**
**p-tau181/np-tau (WU/WU)**	0.410	0.641	0.590	0.708	685	p-tau181 (WU)	0.159	**<0.0001**	**0.00025**
**p-tau181/Aβ40 (WU/WU)**	0.291	0.539	0.476	0.602	685	p-tau181 (WU)	0.0371	**0.021**	**0.032**
p-tau181 (WU)	0.252	0.502	0.437	0.567	685				
**p-tau205/np-tau (WU/WU)**	0.342	0.585	0.522	0.647	653	p-tau205 (WU)	0.0178	0.069	0.093
**p-tau205/Aβ40 (WU/WU)**	0.328	0.573	0.510	0.636	653	p-tau205 (WU)	0.0063	0.31	0.33
p-tau205 (WU)	0.323	0.568	0.505	0.632	653				
**eMTBR-tau243/np-tau (WU/WU)**	0.243	0.493	0.326	0.661	108	eMTBR-tau243 (WU)	0.0535	**0.0009**	**0.0054**
**eMTBR-tau243/Aβ40 (WU/WU)**	0.208	0.456	0.285	0.628	108	eMTBR-tau243 (WU)	0.0190	**0.0088**	**0.017**
eMTBR-tau243 (WU)	0.189	0.435	0.2612	0.608	108				

**Aβ42/Aβ40 (WU/WU)**	0.146	-0.382	-0.457	-0.331	687	Aβ42 (WU)	0.114	**<0.0001**	**0.00025**
Aβ42 (WU)	0.0322	-0.179	-0.253	-0.106	687				
Aβ40 (WU)	0.0102	0.101	0.0265	0.176	687				
np-tau181-190 (WU)	0.0437	0.209	0.136	0.283	685				
np-tau195-210 (WU)	0.0816	0.286	0.212	0.359	653				
np-tau212-221 (WU)	0.0246	0.157	0.0824	0.231	683				

Supplementary Table 8: Intra-individual variance of total variance (IV/TV) for CSF biomarkers in BF1. BF1 IV/TV results for CSF biomarkers p-tau217, p-tau181 and Aβ42 alone or in ratios with Aβ40. IV/TV usually increased for ratios with Aβ40 compared to biomarkers alone. Turquoise represents a ratio with Aβ40 and black the biomarker alone.

Biomarker	Aβ-status	N (# visits)	% Intra variance of total variance (IV/TV)	Compared against	P-value	P-value FDR corrected
**CSF p-tau217 (Li)**	Aβ+	100 (three)<br>37 (four)<br>1 (five)	7.69%			
	Aβ-	213 (three)<br>80 (four)<br>5 (five)	10.0%			
**CSF p-tau217/Aβ40 (Li/El)**	Aβ+	100 (three)<br>37 (four)<br>1 (five)	14.0%	CSF p-tau217 (Li) Aβ+	**<0.001**	**0.0012**
	Aβ-	213 (three)<br>80 (four)<br>5 (five)	16.2%	CSF p-tau217 (Li) Aβ-	**<0.001**	**0.0012**
**CSF p-tau181 (El)**	Aβ+	102 (three)<br>39 (four)<br>1 (five)	5.58%			
	Aβ-	220 (three)<br>82 (four)<br>6 (five)	8.24%			
**CSF p-tau181/Aβ40 (El/El)**	Aβ+	102 (three)<br>39 (four)<br>1 (five)	22.5%	CSF p-tau181 (El) Aβ+	**<0.001**	**0.0012**
	Aβ-	220 (three)<br>82 (four)<br>6 (five)	34.6%	CSF p-tau181 (El) Aβ-	**<0.001**	**0.0012**
**CSF Aβ42 (El)**	Aβ+	102 (three)<br>39 (four)<br>1 (five)	21.2%			
	Aβ-	220 (three)<br>82 (four)<br>6 (five)	21.4%			
**CSF Aβ42/Aβ40 (El/El)**	Aβ+	102 (three)<br>39 (four)<br>1 (five)	16.5%	CSF Aβ42 (El) Aβ+	**0.035**	**0.035**
	Aβ-	220 (three)<br>82 (four)<br>6 (five)	30.0%	CSF Aβ42 (El) Aβ-	**<0.001**	**0.0012**

Supplementary Table 9: Linear mixed effects models for CSF biomarker changes over time in BF1. Linear mixed effects models with independent variables time, Aβ-status and time x Aβ-status were fitted to the longitudinal BF1 biomarker data (dependent variable) CSF biomarkers p-tau217, p-tau181 and Aβ42 alone or in ratios with Aβ40. The Aβ40 ratio reduced participant variance by 39-80% and increased Aβ-status differentiation, but biomarker changes over time were only minimally affected by the ratio approach. Turquoise represents a ratio with Aβ40 and black the biomarker alone.

**Outcome**	**N (# visits)**	**Aβ-status coeff**	**Aβ-status P-value**	**Aβ-status x time coeff**	**Aβ-status x time P-value**	**Time coeff**	**Time P-value**	**Participant variance**	**Participant P-value**	**Log-likelihood**
**CSF p-tau217 (Li)**	579 (two)<br>337 (three)<br>132 (four)<br>12 (five)	0.263	3.70e-08	0.00546	1.94e-23	0.000643	0.0393	14.7	2.08e-33	-1070
**CSF p-tau217/Aβ40 (Li/El)**	579 (two)<br>337 (three)<br>132 (four)<br>12 (five)	0.394	1.32e-11	0.00662	5.64e-22	0.000644	0.100	8.07	1.03e-29	-1280
**CSF p-tau181 (El)**	587 (two)<br>346 (three)<br>137 (four)<br>13 (five)	0.232	1.19e-08	0.00297	4.08e-10	0.00187	6.40e-12	20.5	1.65e-37	-973
**CSF p-tau181/Aβ40 (El/El)**	587 (two)<br>346 (three)<br>137 (four)<br>13 (five)	0.620	2.98e-21	0.00404	8.38e-06	0.00154	3.24e-03	4.02	4.32e-30	-1620
**CSF Aβ42 (El)**	587 (two)<br>346 (three)<br>137 (four)<br>13 (five)	-0.907	3.49e-49	-0.00171	0.0790	0.00141	0.0125	2.53	3.23e-31	-1640
**CSF Aβ42/Aβ40 (El/El)**	587 (two)<br>346 (three)<br>137 (four)<br>13 (five)	-1.53	4.40e-226	-0.000290	0.672	0.000699	0.0793	1.54	3.22e-17	-946

Supplementary Table 10: Linear mixed effects models modeling plasma biomarker change over time in BF1. Linear mixed effects models with independent variables time, Aβ-status and time x Aβ status were fitted to the longitudinal BF1 biomarker data (dependent variable) plasma biomarkers p-tau217 and Aβ42 alone or in ratios with Aβ40 or Aβ42. Turquoise represents a ratio with Aβ40 and black the biomarker alone.

Outcome	N (# visits)	Aβ-status coeff	Aβ-status P-value	Aβ-status x time coeff	Aβ-status x time P-value	Time coeff	Time P-value	Participant variance	Participant P-value	Log-likelihood
**Plasma p-tau217 (Li)**	279 (two)<br>136 (three)<br>42 (four)	0.249	0.0212	0.0116	8.68e-12	-0.000030	0.975	2.95	3.13e-13	-812
**Plasma p-tau217/Aβ40 (Li/WU)**	279 (two)<br>136 (three)<br>42 (four)	0.203	0.0423	0.00963	7.13e-12	-0.00262	7.35e-04	5.20	2.14e-14	-741
**Plasma Aβ42 (WU)**	313 (two)<br>158 (three)<br>47 (four)	-0.478	7.76e-07	0.000253	0.889	0.00904	6.27e-18	1.86	5.35e-16	-983
**Plasma Aβ42/Aβ40 (WU/WU)**	313 (two)<br>158 (three)<br>47 (four)	-0.863	4.90e-21	-0.00181	0.297	-0.00213	0.0346	1.43	6.30e-13	-919
