# Supplementary material for: Reference proteins to improve Core 1 and Core 2 Alzheimer’s disease CSF and plasma biomarkers
Source: Brain. 2025 Oct 6;149(4):1153–67. doi: 10.1093/brain/awaf375 (PMC13058454; doi:10.1093/brain/awaf375)
Supplement: awaf375_Supplementary_Data [file awaf375_supplementary_data.zip › brain-2025-01206-File010.pdf]

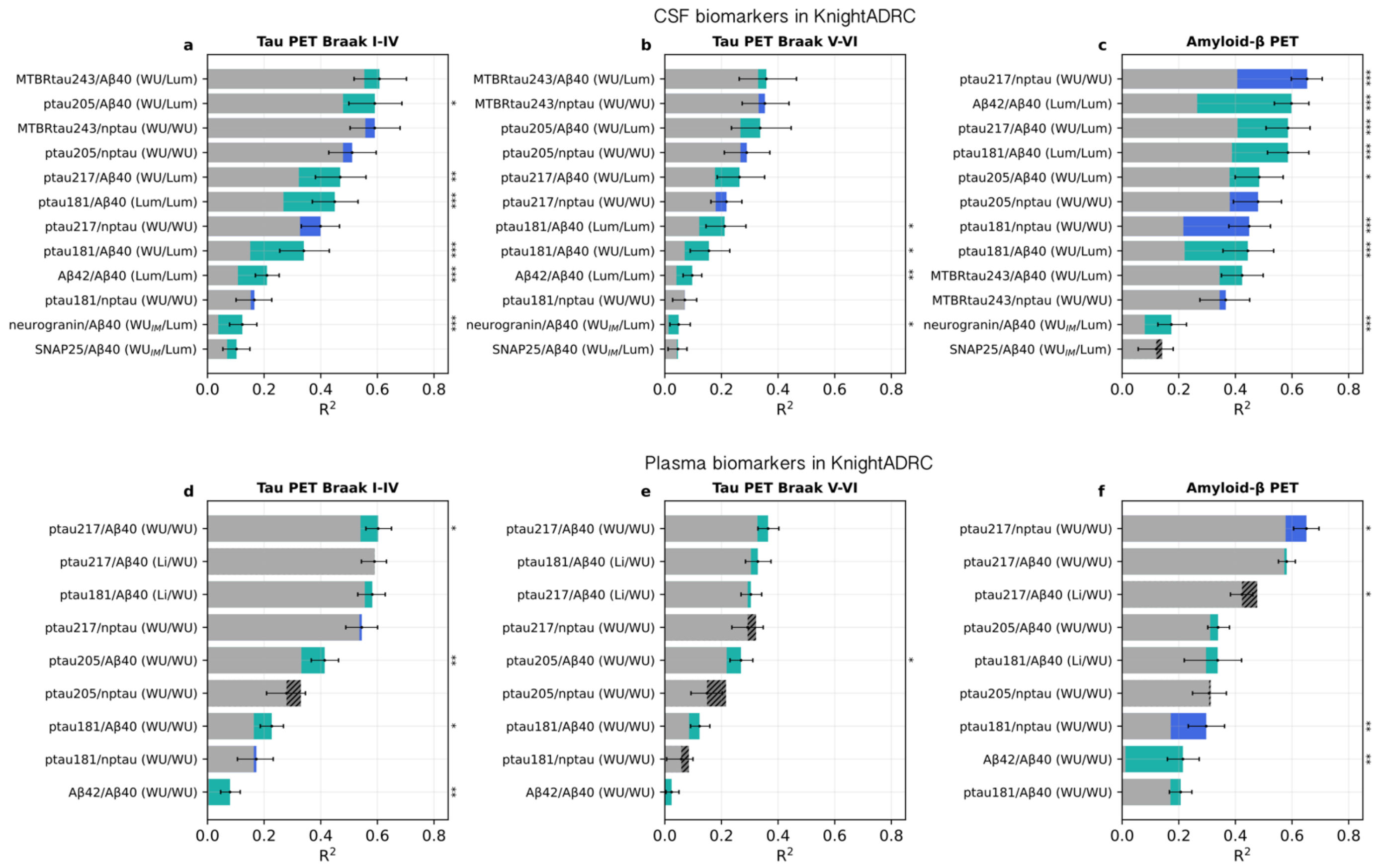

Supplementary Figure 1: Reference protein normalized plasma and CSF biomarkers show stronger correlations with tau and Aβ-PET in Knight ADRC. Horizontal bar-plots showing the proportion of explained variance (R^2) by the biomarker or biomarker ratio for univariate linear regression models with continuous tau or Aβ-PET as outcomes in Knight ADRC. CSF biomarkers in a)-c), and plasma biomarkers in d)-f). Gray bars represent the unnormalized biomarker, turquoise stacked bars improvement from a ratio with Aβ40, blue stacked bars improvement from a ratio with np-tau, and black striped stacked bars when normalization resulted in an R^2 reduction instead of increase. Error bars represent 95% confidence intervals. WU (Washington University, mass spectrometry), WU_{IM} (Washington University, immunoassay), Li (Lilly, immunoassay), Lumipulse (Lum, immunoassay).

*$P < 0.05$, **$P < 0.01$, ***$P < 0.001$ compared against the biomarker alone (assessed with bootstrapping and FDR corrected).

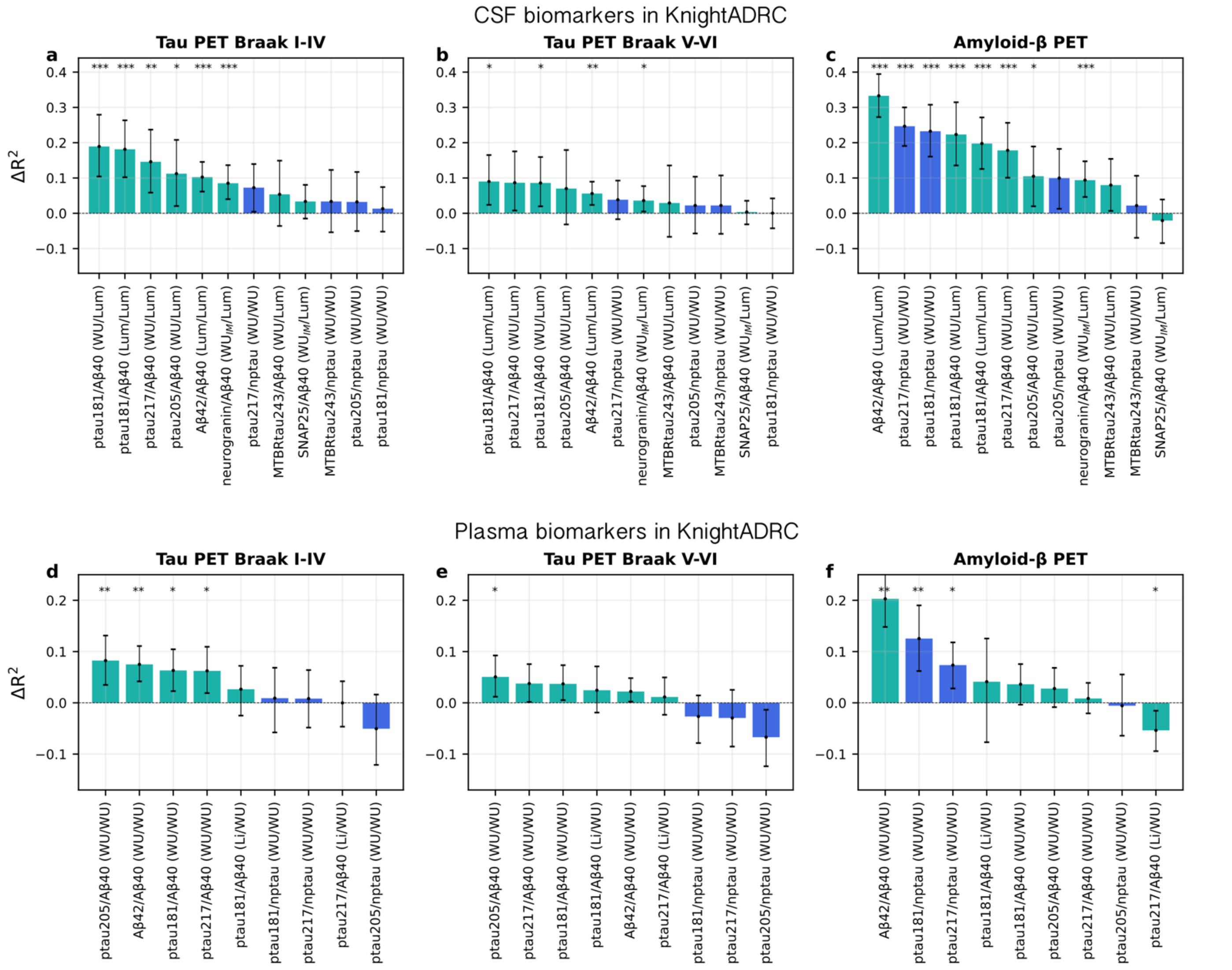

Supplementary Figure 2: R^2 change during reference protein normalization for plasma and CSF biomarkers correlations with tau and Aβ-PET in Knight ADRC. Bar-plots showing the difference in proportion of explained variance (ΔR^2) comparing a normalized biomarker to the same biomarker alone in associations with continuous tau or Aβ-PET in Knight ADRC. CSF biomarkers in a)-c), and plasma biomarkers in d)-f). Turquoise bars represent a ratio with Aβ40 and blue with non-phosphorylated tau. Error bars represent 95% confidence intervals. Further details on all models can be seen in Supplementary Knight ADRC.

Abbreviations: WU (Washington University, mass spectrometry), WU_{IM} (Washington University, immunoassay), Li (Lilly, immunoassay), Lumipulse (Lum, immunoassay).

**P* < 0.05, ***P* < 0.01, ****P* < 0.001 compared against the biomarker alone (assessed with bootstrapping and FDR corrected).

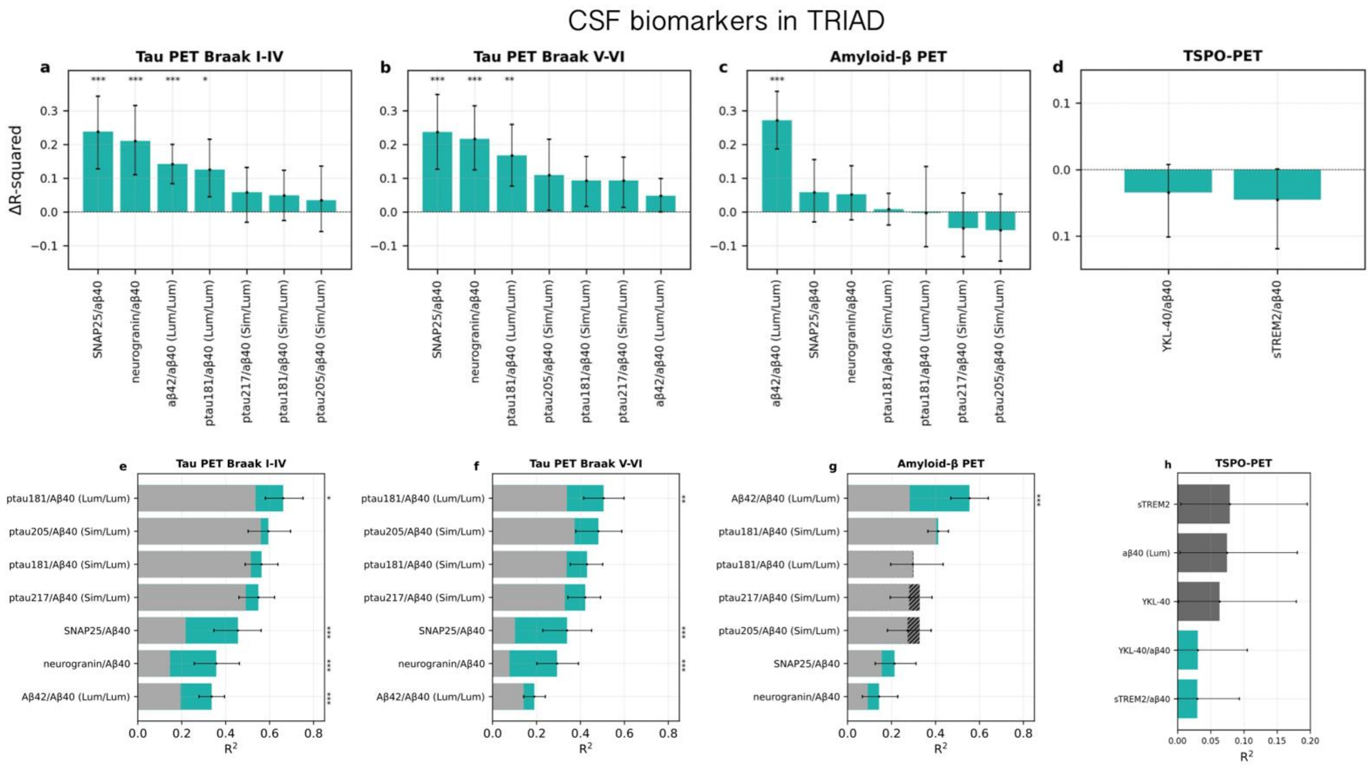

Supplementary Figure 3: Core AD and synaptic CSF biomarker show stronger associations with tau and Aβ-PET with Aβ40 normalization, but not inflammatory CSF biomarkers with TSPO-PET in TRIAD. a)-d) Bar-plots showing the difference in proportion of explained variance (ΔR^2) between a biomarker ratio with Aβ40 versus the biomarker alone. The analyses are univariate linear regression models with continuous tau, amyloid or TSPO PET as outcomes. e)-h) Stacked R^2 bar plots of normalized and non-normalized biomarkers in TRIAD for associations with continuous tau-PET, Aβ-PET or TSPO-PET. Turquoise bars represent a ratio with Aβ40 and gray the biomarker alone. Error bars represent 95% confidence intervals.

Abbreviations: Lum (Lumipulse, immunoassay), Sim (Simoa)

**P* < 0.05, ***P* < 0.01, ****P* < 0.001 compared against the biomarker alone (assessed with bootstrapping and FDR corrected).

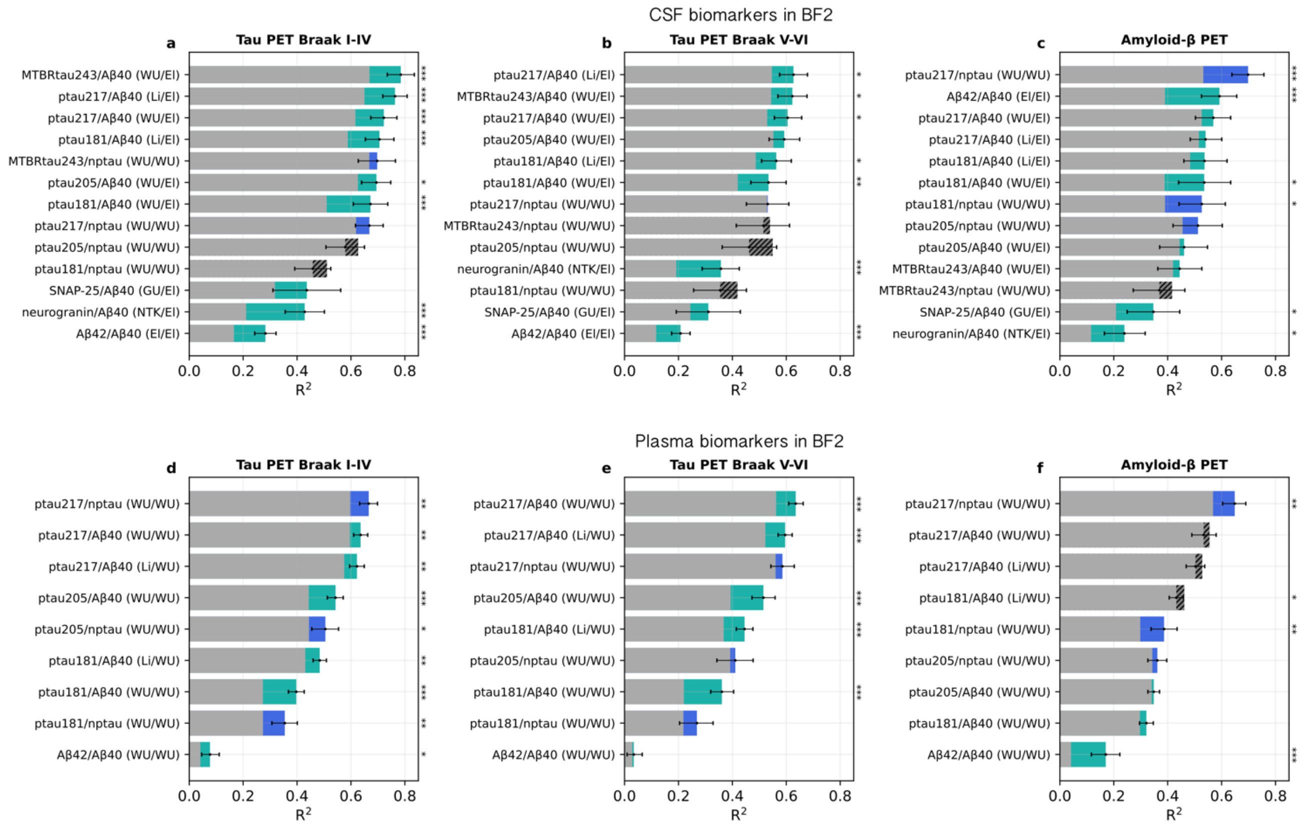

Supplementary Figure 4: Head-to-head comparison of normalized and unnormalized fluid biomarkers in a subset of participants with all data available in BF2. The change in R^2 during reference protein normalization for fluid biomarker associations with continuous tau or Aβ-PET in BF2. Gray bars represent the unnormalized biomarker, turquoise stacked bars improvement from a ratio with Aβ40, blue stacked bars improvement from a ratio with np-tau, and black striped stacked bars when normalization resulted in an R^2 reduction instead of improvement. Analyses were made on a subsample of BF2 with complete data only: n=322 in a) and b), n=193 in c), n=875 in d) and e), n=602 in f). Note that plasma MTBR-tau243 was excluded from this analysis due to plasma samples being from a different visit than the other plasma biomarkers (resulting in no overlap of participants with all data available).

Abbreviations: WU (Washington University, mass spectrometry), GU (University of Gothenburg, mass spectrometry), Li (Lilly, immunoassay), El (Elecsys, immunoassay), NTK (NeuroToolKit, immunoassay).

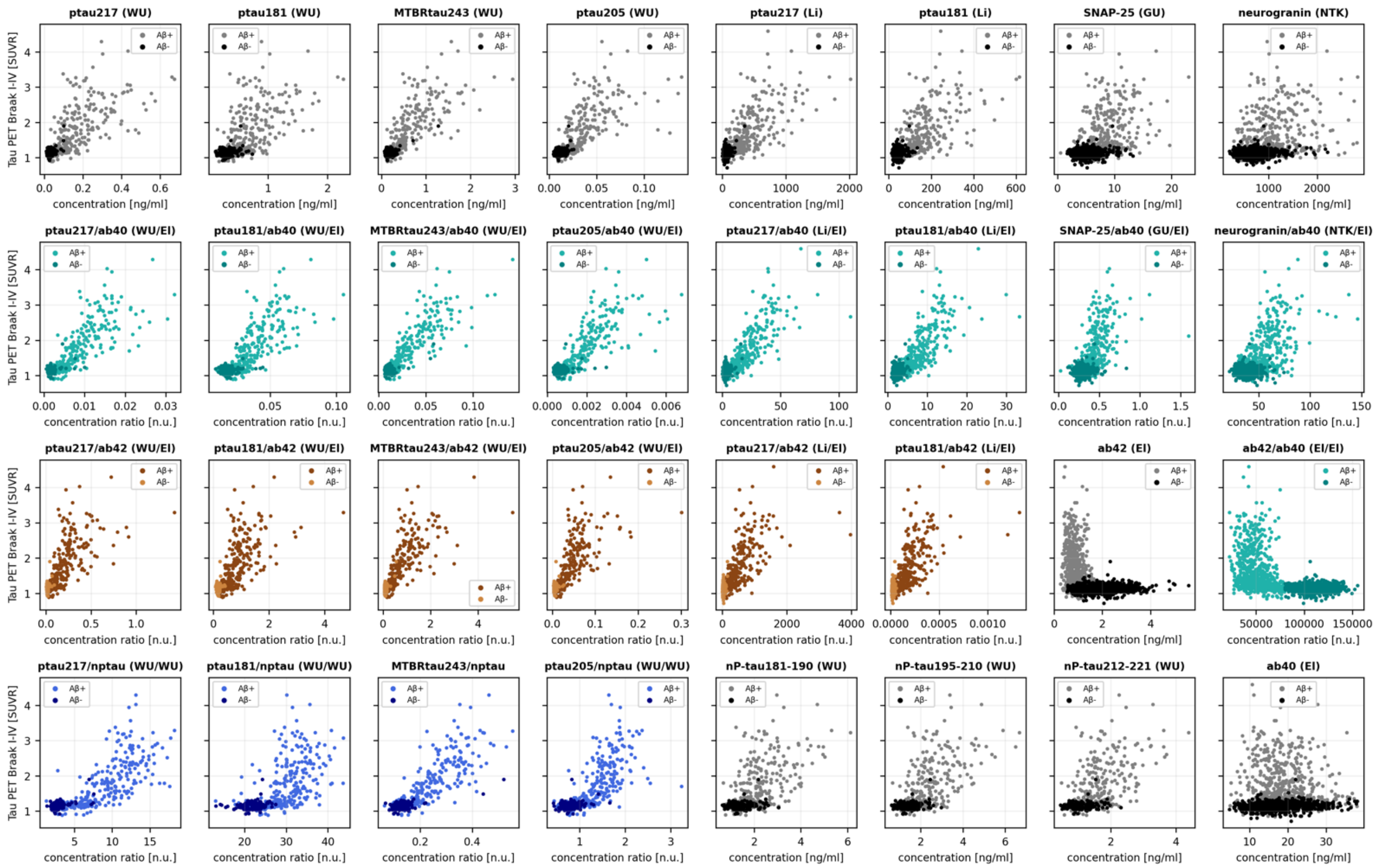

Supplementary Figure 5: Scatter plots for all tested CSF biomarkers and ratios against tau PET Braak I-IV in BF2. Turquoise plots represent a ratio with Aβ40, blue with non-phosphorylated tau, orange with Aβ42 and black/gray the biomarker alone. Details on corresponding univariate linear models can be seen in Table 1.

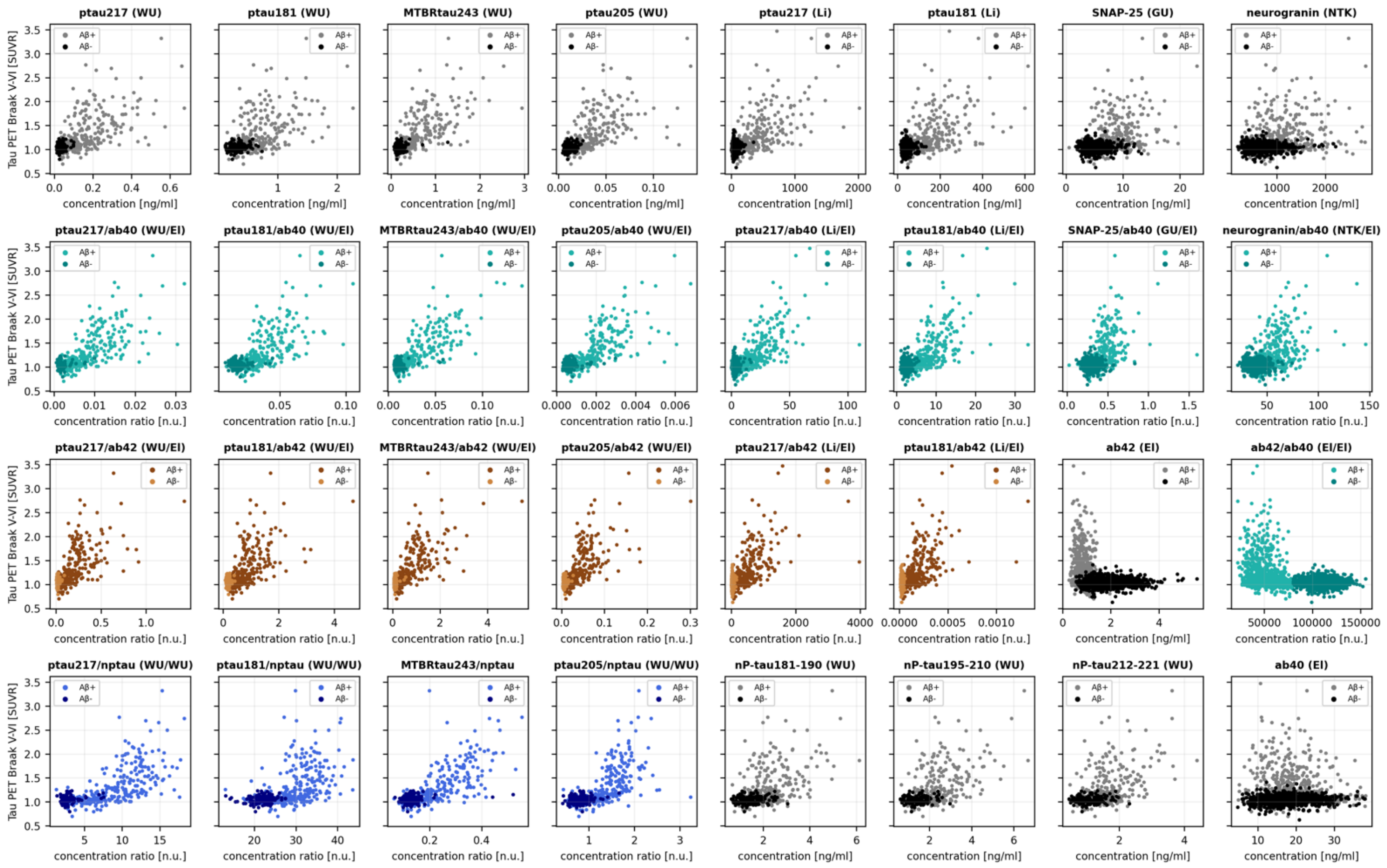

Supplementary Figure 6: Scatter plots for all tested CSF biomarkers and ratios against tau PET Braak V-VI in BF2. Turquoise plots represent a ratio with Aβ40, blue with non-phosphorylated tau, orange with Aβ42 and black/gray the biomarker alone. Details on corresponding univariate linear models can be seen in Supplementary Table 2.

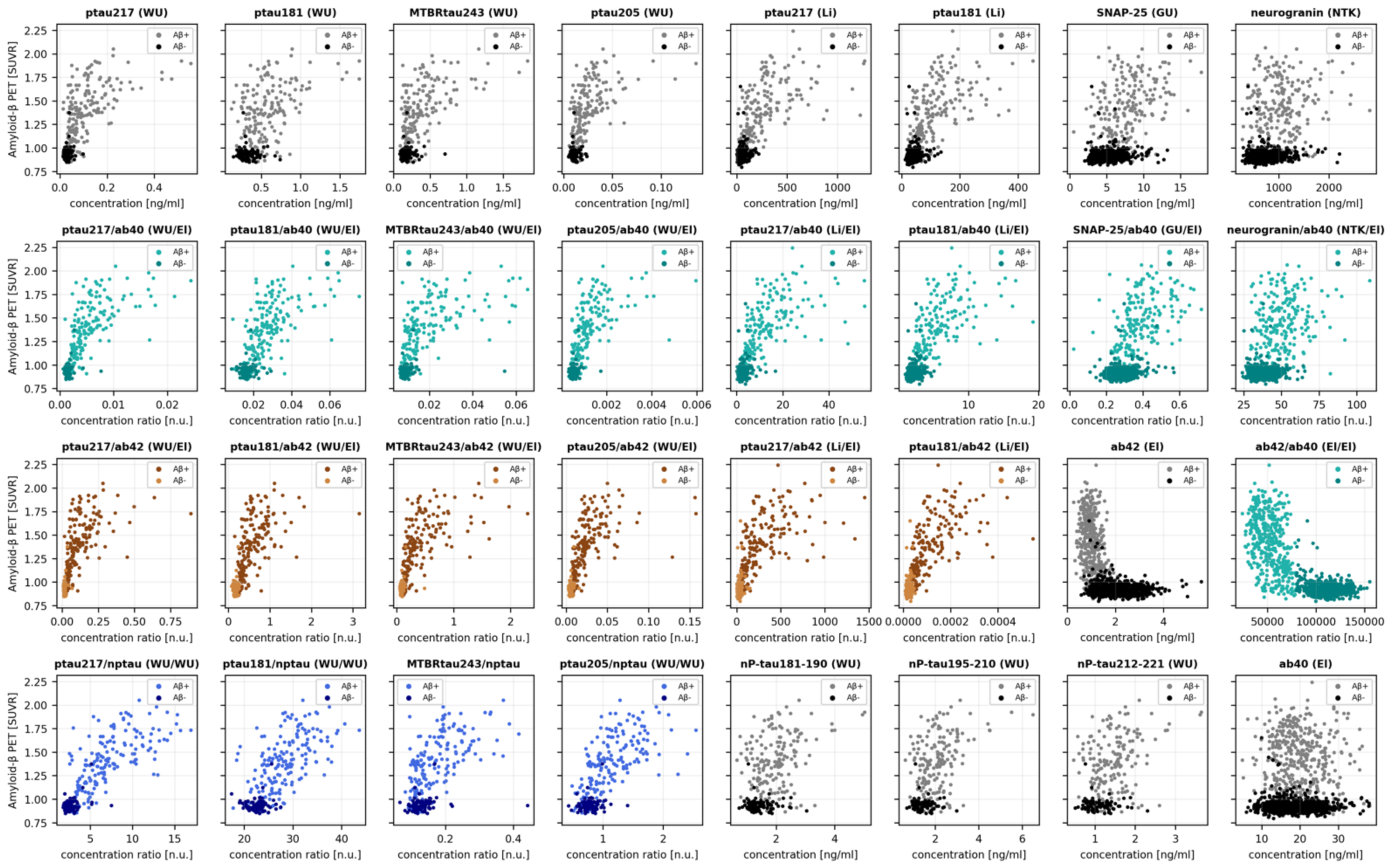

Supplementary Figure 7: Scatter plots for all tested CSF biomarkers and ratios against Aβ-PET in BF2. Turquoise plots represents a ratio with Aβ40, blue with non-phosphorylated tau, orange with Aβ42 and black/gray the biomarker alone. Details on corresponding univariate linear models can be seen in Supplementary Table 4.

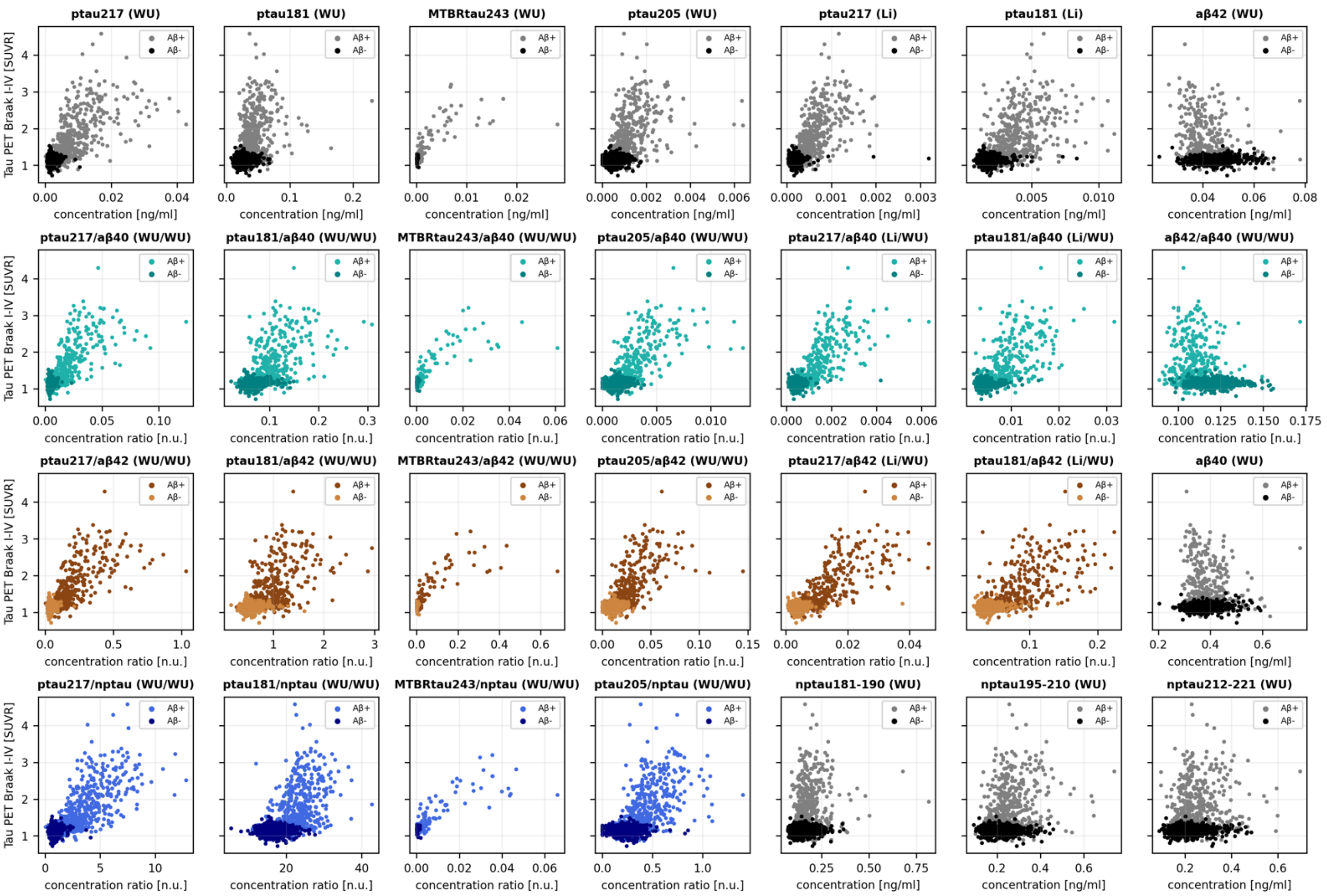

Supplementary Figure 8: Scatter plots for all tested plasma biomarkers and ratios against tau PET Braak I-IV in BF2. Turquoise plots represent a ratio with Aβ40, blue with non-phosphorylated tau, orange with Aβ42 and black/gray the biomarker alone. Details on corresponding univariate linear models can be seen in Supplementary Table 5.

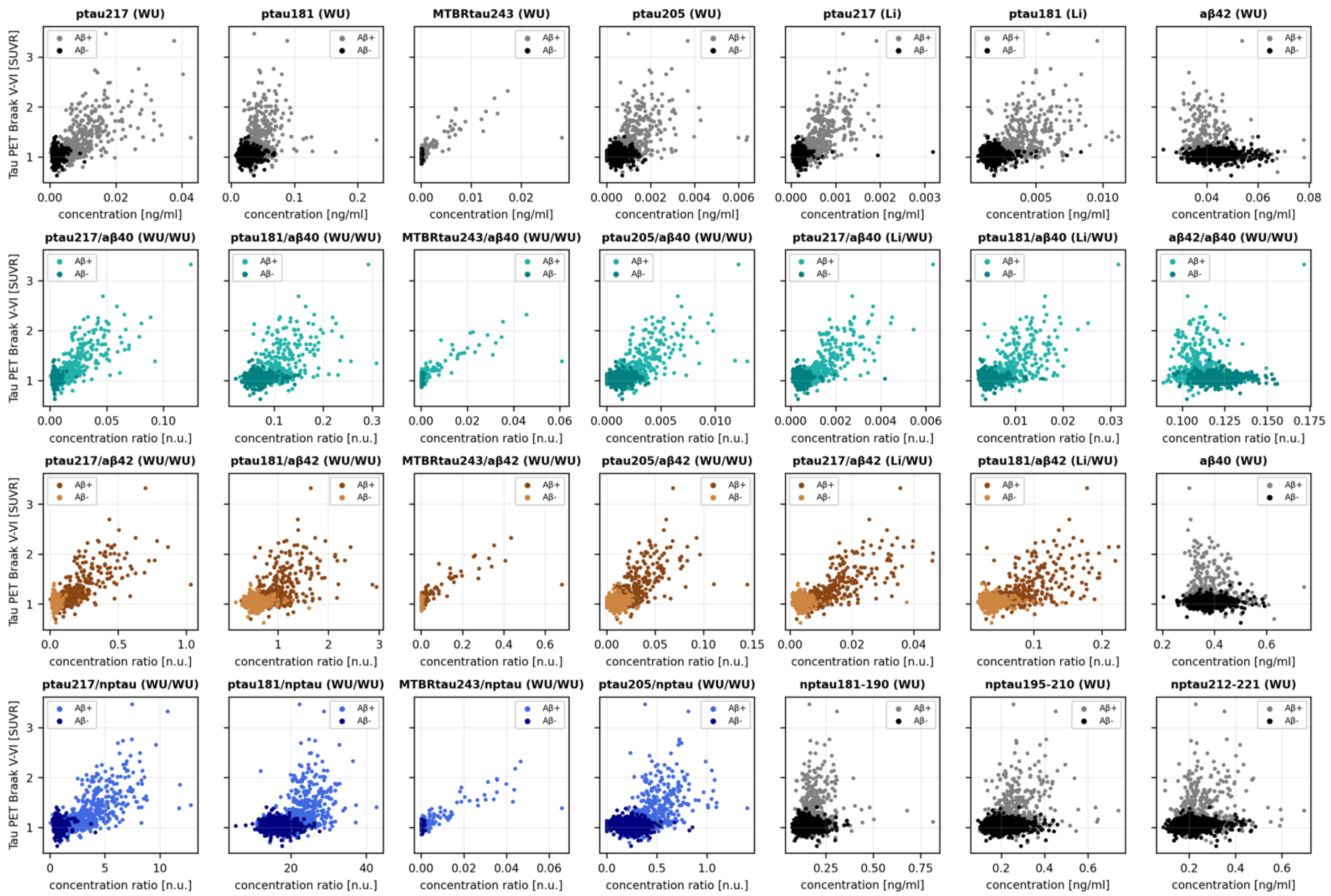

Supplementary Figure 9: Scatter plots for all tested plasma biomarkers and ratios against tau PET Braak V-VI in BF2. Turquoise plots represent a ratio with Aβ40, blue with non-phosphorylated tau, orange with Aβ42 and black/gray the biomarker alone. Details on corresponding univariate linear models can be seen in Supplementary Table 6.

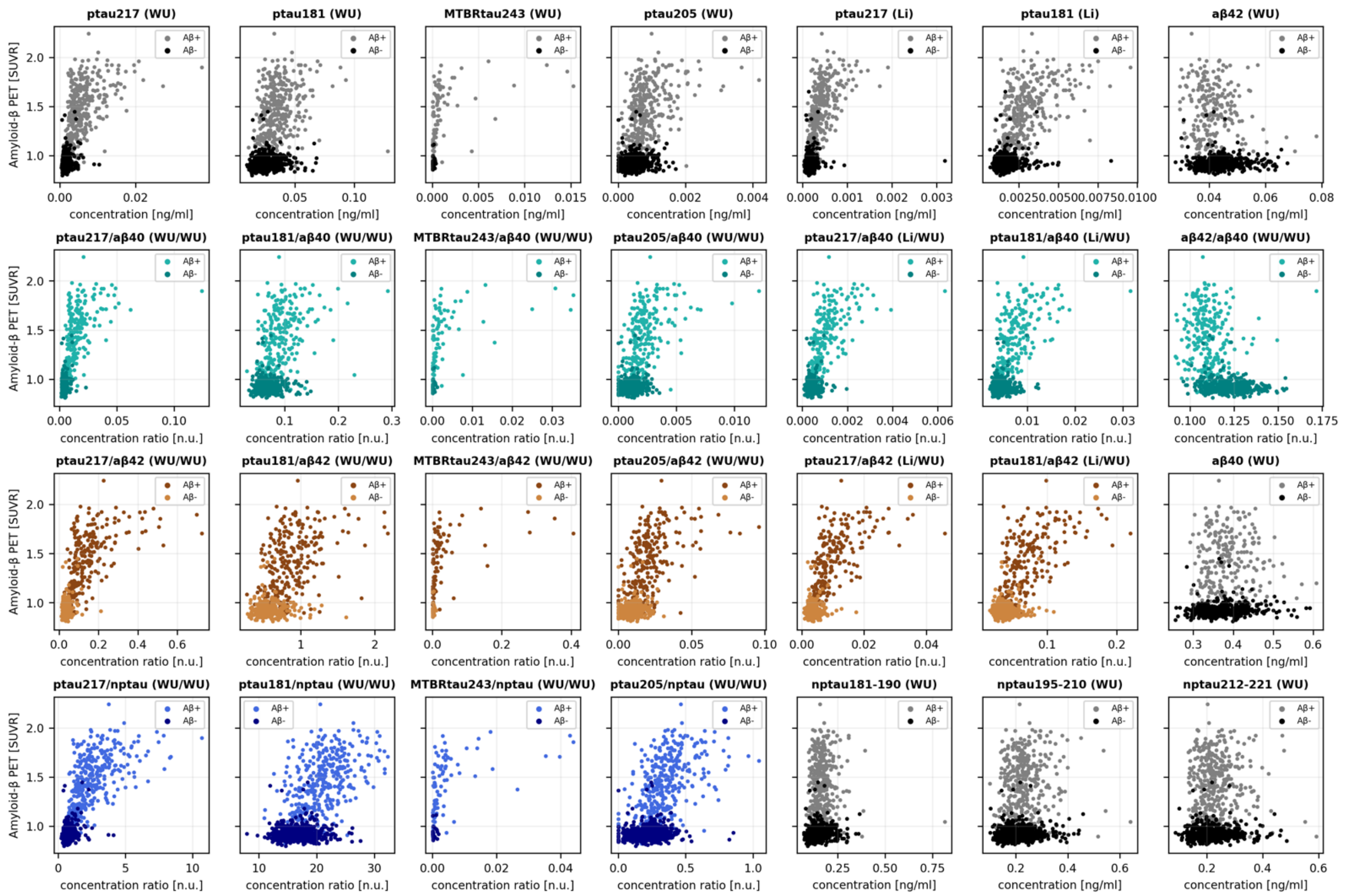

Supplementary Figure 10: Scatter plots for all tested plasma biomarkers and ratios against Aβ-PET in BF2. Turquoise plots represent a ratio with Aβ40, blue with non-phosphorylated tau, orange with Aβ42 and black/gray the biomarker alone. Details on corresponding univariate linear models can be seen in Supplementary Table 8.

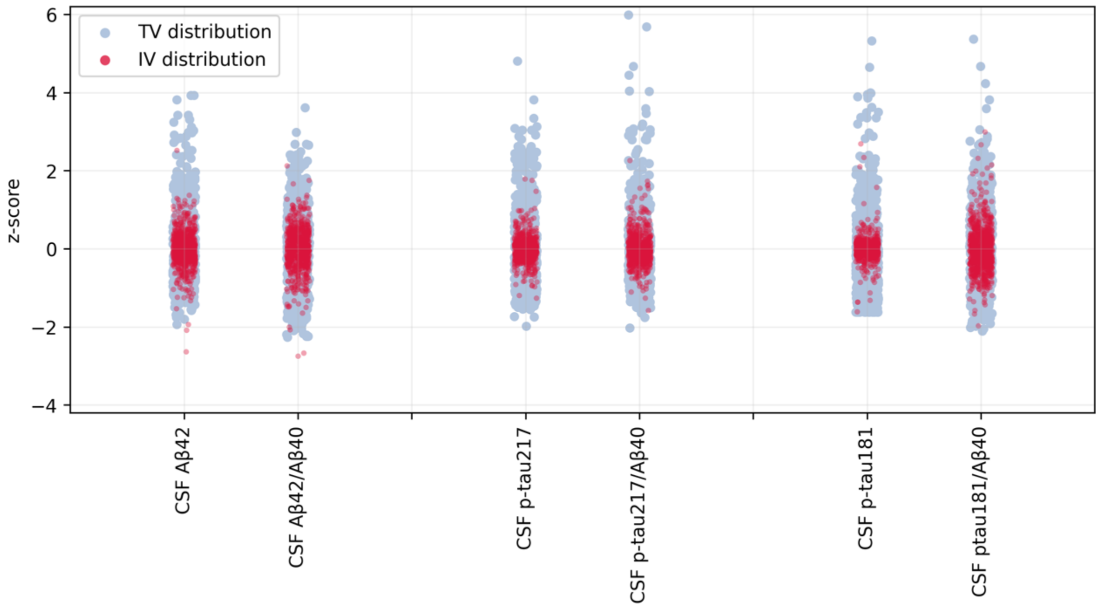

Supplementary Figure 11: Distribution of intra-individual variance compared to total variance (IV/TV) for CSF biomarkers in BF1 Aβ-negative individuals. Distribution of IV (red) and TV (gray) in BF1 Aβ-negative individuals for CSF biomarkers Aβ42, p-tau217 and p-tau181 alone or in ratios with Aβ40. IV/TV usually increased for ratios with Aβ40 compared to biomarkers alone.

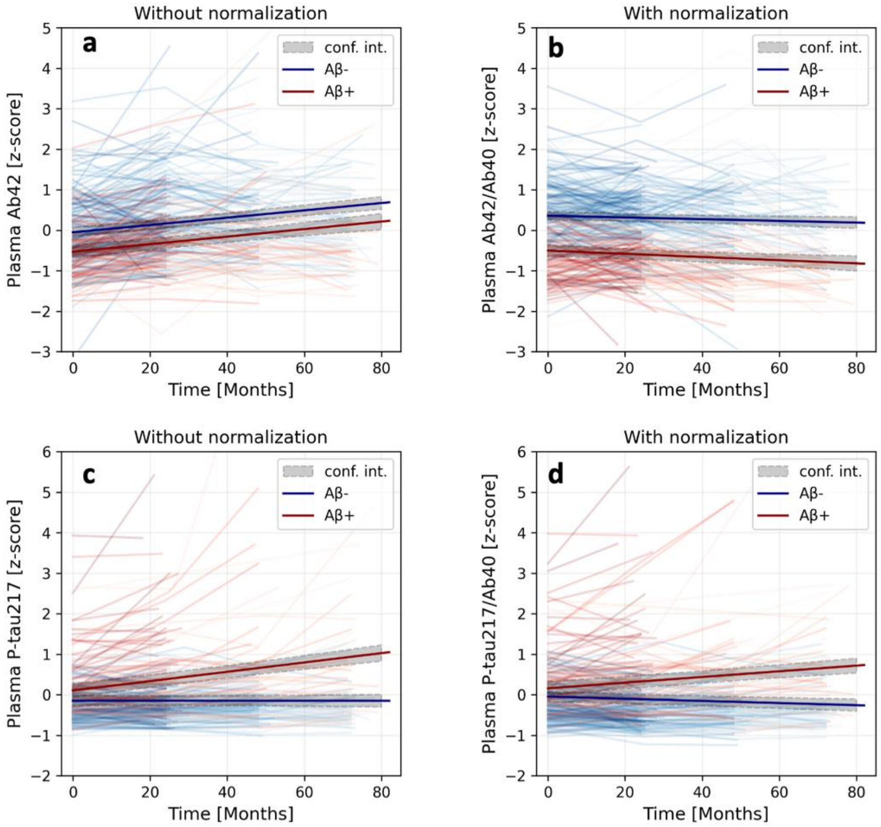

Supplementary Figure 12: Normalized plasma biomarkers show a similar change over time as biomarkers alone, but with reduced inter-individual variance. Linear mixed models with independent variables time, Aβ-status and time x Aβ-status were fitted to the longitudinal BF1 biomarker data (dependent variable) for a) plasma Aβ42 (WU) and c) p-tau217 (El), and corresponding markers in a ratio with plasma Aβ40 or Aβ42 (WU) in b), d) and e). Aβ-positive in red and Aβ-negative in blue. Further details on all models can be seen in Supplementary Table 11.

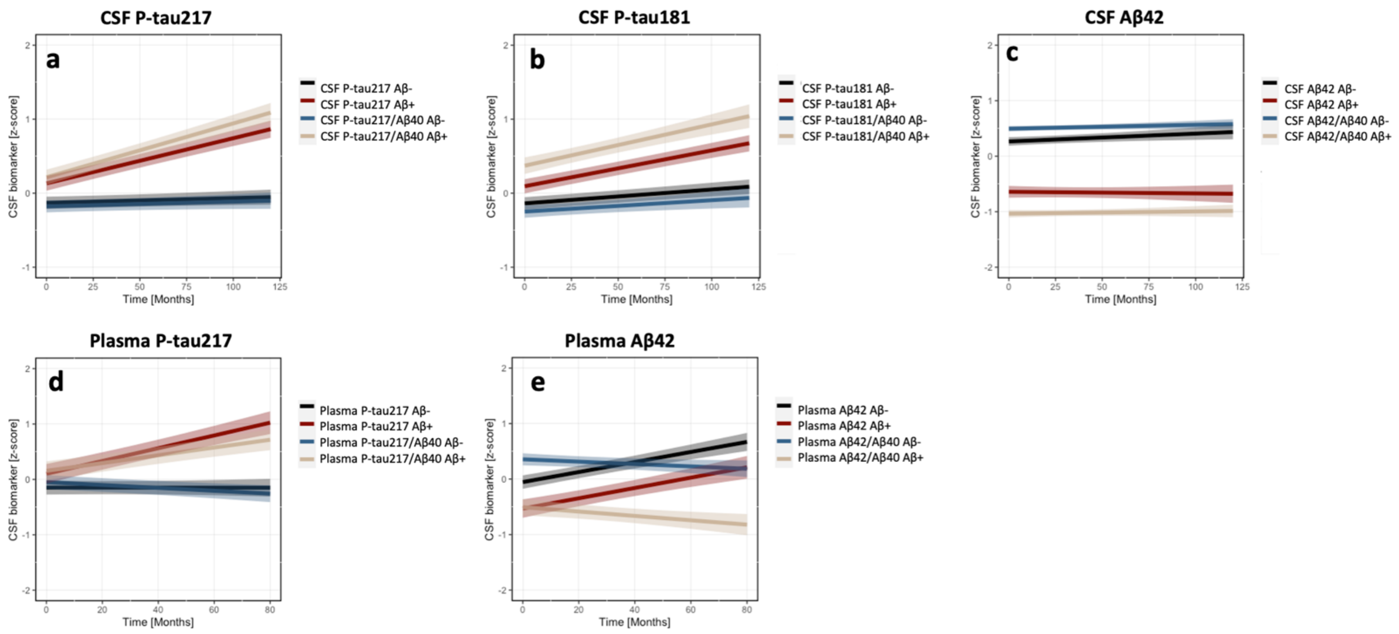

Supplementary Figure 13: Merged version of biomarker comparisons in Figure 4 and Supplementary Figure 11. Linear mixed models with independent variables time, Aβ-status and time x Aβ-status were fitted to the longitudinal BF1 biomarker data (dependent variable) and ratio with CSF/plasma Aβ40 (El/WU) for a) CSF p-tau217 (Li), b) CSF p-tau181 (El), c) CSF Aβ42 (El), d) plasma p-tau217 (El) and e) plasma Aβ42 (WU).

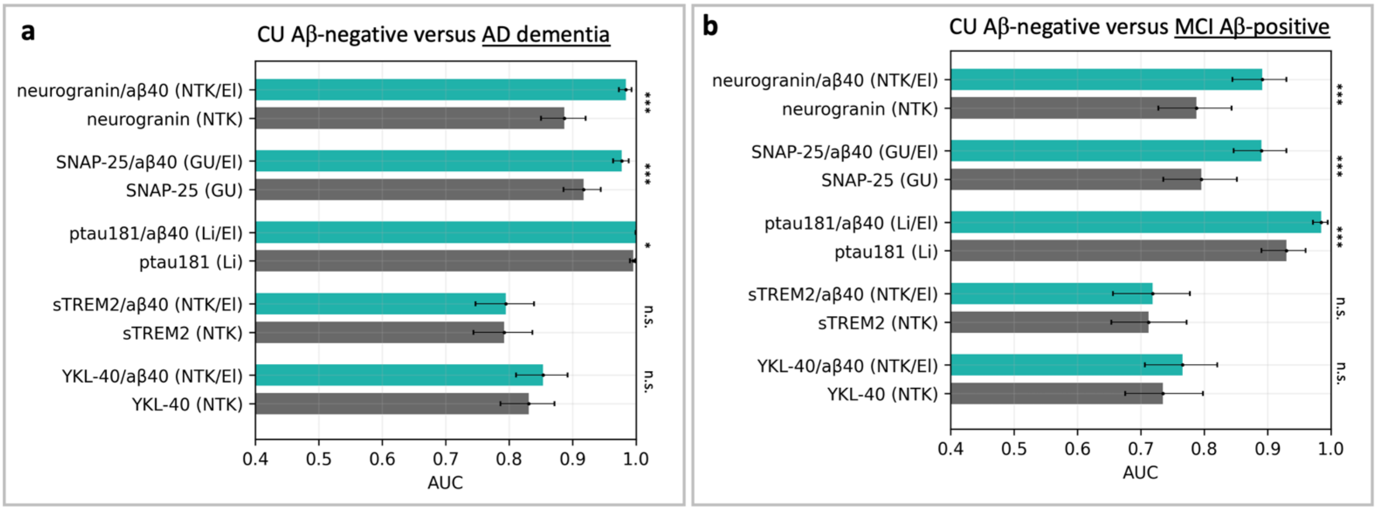

Supplementary Figure 14: Group level differences comparing AD and inflammatory CSF biomarkers with and without CSF Aβ40 normalization. Logistic regression models with outcomes a) CU Aβ-negative versus AD dementia (n=354, 247 negative and 107 positive) and b) CU Aβ-negative versus MCI Aβ-positive (n=318, 251 negative and 67 positive). AD biomarkers neurogranin, SNAP-25 and p-tau181 increased significantly in AUC when in a ratio with CSF Aβ40, while no significant difference was seen for inflammatory markers sTREM2 and YKL-40. We chose to include neurogranin, SNAP-25 and p-tau181 as AD biomarkers in these analyses due to their relatively low AUC without a reference protein, leaving room for improvement. CSF p-tau217 and MTBR-tau243 for example already distinguished the groups with AUCs close to 1, reducing the relevance of using a reference protein normalization.

*$P < 0.05$, **$P < 0.01$, ***$P < 0.001$ compared against the biomarker alone (assessed with bootstrapping and FDR corrected).

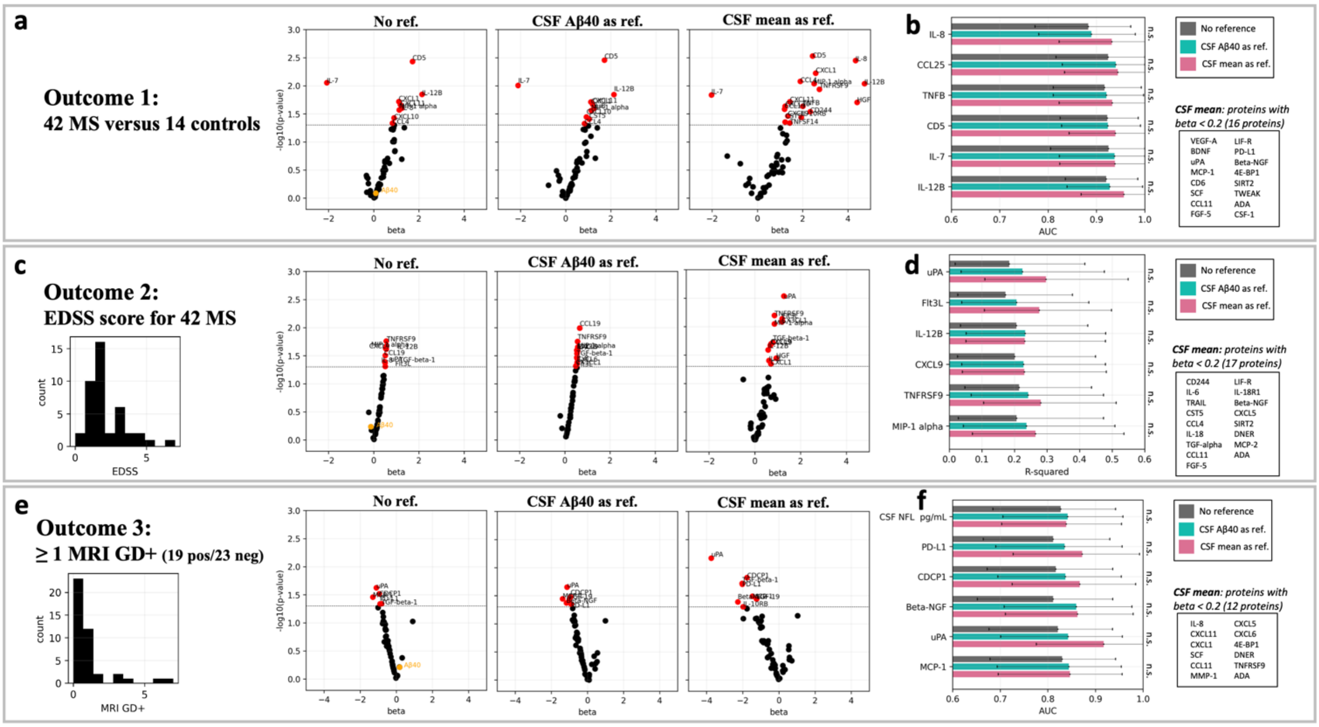

Supplementary Figure 15: Exploring the broader applicability of CSF Aβ40 as a reference protein by comparing differentially expressed CSF inflammatory OLINK proteins in an MS cohort. a) MS versus controls (other neurodegenerative diseases) in a logistic regression model with a CSF OLINK protein as main predictor, and age, sex and (if specified) a reference protein as covariates. Aβ40 showed low correlation with the outcome but only minor effects were seen when using it as a reference protein in both change in a) associative strength and b) AUC for the six proteins most strongly associated with the outcome. A larger improvement was generally observed when instead adjusting for a mean CSF level created from the proteins with lowest correlation to the outcome (absolute beta-coefficient < 0.2). Similar results were seen in a linear regression model with EDSS score as outcome (c and d), and for the binary outcome ≥ MRI gadolinium (GD) lesions in a logistic regression model (e and f). These two analyses were performed only on the MS participants and included adjustment for age, sex and (if specified) a reference protein. Note that the sample size in this cohort was small and no AUC findings were significant.
